# Aerosol delivery during invasive mechanical ventilation: a systematic review

**DOI:** 10.1186/s13054-017-1844-5

**Published:** 2017-10-21

**Authors:** Jonathan Dugernier, Stephan Ehrmann, Thierry Sottiaux, Jean Roeseler, Xavier Wittebole, Thierry Dugernier, François Jamar, Pierre-François Laterre, Gregory Reychler

**Affiliations:** 1 Institut de Recherche Expérimentale et Clinique (IREC), Pneumologie, ORL & Dermatologie, Cliniques universitaires Saint-Luc, Avenue Hippocrate 10, 1200 Brussels, Belgium; 2 Soins Intensifs, Cliniques universitaires Saint-Luc, Avenue Hippocrate 10, 1200 Brussels, Belgium; 3 Médecine Physique, Cliniques universitaires Saint-Luc, Avenue Hippocrate 10, 1200 Brussels, Belgium; 4 Université François Rabelais, UMR 1100, F-37032 Tours, France; 5 INSERM, Centre d’étude des Pathologies Respiratoires, UMR 1100, F-37032 Tours, France; 6 CHRU de Tours, Réanimation polyvalente, F-37044 Tours, France; 7 Soins Intensifs, Clinique Notre-Dame de Grace, Chaussée de Nivelles 212, 6041 Charleroi, Belgium; 8 Soins Intensifs, Clinique Saint-Pierre, Avenue Reine Fabiola 9, 1340 Ottignies, Belgium; 9 Médecine Nucléaire, Cliniques universitaires Saint-Luc, Avenue Hippocrate 10, 1200 Brussels, Belgium; 10 Pneumologie, Cliniques universitaires Saint-Luc, Avenue Hippocrate 10, 1200 Brussels, Belgium

**Keywords:** Nebulizer, Antibiotics, Bronchodilators, Scintigraphy

## Abstract

**Background:**

This systematic review aimed to assess inhaled drug delivery in mechanically ventilated patients or in animal models. Whole lung and regional deposition and the impact of the ventilator circuit, the artificial airways and the administration technique for aerosol delivery were analyzed.

**Methods:**

In vivo studies assessing lung deposition during invasive mechanical ventilation were selected based on a systematic search among four databases. Two investigators independently assessed the eligibility and the risk of bias.

**Results:**

Twenty-six clinical and ten experimental studies were included. Between 30% and 43% of nominal drug dose was lost to the circuit in ventilated patients. Whole lung deposition of up to 16% and 38% of nominal dose (proportion of drug charged in the device) were reported with nebulizers and metered-dose inhalers, respectively. A penetration index inferior to 1 observed in scintigraphic studies indicated major proximal deposition. However, substantial concentrations of antibiotics were measured in the epithelial lining fluid (887 (406–12,819) μg/mL of amikacin) of infected patients and in sub-pleural specimens (e.g., 197 μg/g of amikacin) dissected from infected piglets, suggesting a significant distal deposition. The administration technique varied among studies and may explain a degree of the variability of deposition that was observed.

**Conclusions:**

Lung deposition was lower than 20% of nominal dose delivered with nebulizers and mostly occurred in proximal airways. Further studies are needed to link substantial concentrations of antibiotics in infected pulmonary fluids to pulmonary deposition. The administration technique with nebulizers should be improved in ventilated patients in order to ensure an efficient but safe, feasible and reproducible technique.

**Electronic supplementary material:**

The online version of this article (doi:10.1186/s13054-017-1844-5) contains supplementary material, which is available to authorized users.

## Background

Aerosol therapy is commonly used in the intensive care unit. Three primary classes of drugs are delivered by inhalation to mechanically ventilated patients: bronchodilators, corticosteroids and antibiotics [1]. Drug efficacy depends on the dose and the site of deposition. The clinical benefit of bronchodilators plateaus the effective dose is deposited, and increasing the dose will expose the patient to potential adverse events (e.g., cough, tachycardia and tremor) [2, 3]. With respect to inhaled antibiotics, concentrations should be maximized at the infected lung site to obtain an effective bactericidal effect according to their pharmacokinetics-pharmacodynamics characteristics [4].

Many factors influence aerosol delivery to the lungs during mechanical ventilation and are related to the drug, the device, the patient, the ventilator circuit, the artificial airways and the ventilator settings [5]. These factors have been primarily studied in vitro [6–8]. During the past 30 years, clinical and experimental studies have investigated lung deposition of inhaled drugs during invasive mechanical ventilation using imaging techniques based on radiolabeled aerosol deposition, lung tissue sampling and pharmacokinetics analysis [5, 9, 10]. A comprehensive systematic review summarizing in vivo data related to aerosol delivery during invasive mechanical ventilation has never been published.

The aim of this review is to evaluate studies that assessed in vivo lung delivery of inhaled drugs to mechanically ventilated patients or animal models either as absolute drug concentrations or quantitative deposition relative to the nominal dose to: (1) provide current knowledge on whole lung deposition; (2) examine the distribution and penetration of inhaled drugs into different regions of the respiratory tract; (3) determine how the ventilator circuit and the artificial airways impact aerosol delivery and (4) discuss the administration techniques applied in these studies.

## Methods

This study was registered in the International Prospective Register of Systematic Reviews (PROSPERO CRD42016047186) and was conducted according to the Preferred Reporting Items for Systematic Reviews and Meta-Analyses (PRISMA) guidelines [11].

### Search strategy and data extraction

The search strategy, selection criteria, data extraction and study quality assessment are detailed in Additional file 1. The systematic search was performed among the Pubmed, Science Direct, Scopus and PeDRO database by one investigator (JD) who examined publications from 1985 to Sept 2016. Original research articles were included according to inclusion criteria based on participants, interventions, comparisons, outcomes and study design (PICOS) (Table 1). Articles published in a language other than English and French, were excluded. The Downs and Black scale was used to define the methodological quality of eligible studies [12].

**Table 1:** Inclusion criteria for studies according PICOS

Participants	Adult aged 18 years or older with invasive mechanical ventilation
	or in vivo experimental model of adult invasive mechanical ventilation
Interventions	Aerosol administration using any type of device (nebulizer, metered-dose inhaled, dry powder inhaler, etc.)
	Aerosol deposition assessment using pharmacokinetics or radioisotopic methods
Outcomes	Pulmonary deposition of inhaled drug (dose, distribution or penetration)
	Extrapulmonary deposition, if available
Study designs	RCT, randomized comparative, crossover or cohort studies

*PICOS* participants, interventions, comparisons, outcomes and study design, *RCT* randomized controlled trial

### Data expression

The data were expressed as the mean ± standard deviation or median (25–75% interquartile range). Lung deposition data were expressed as percentage of nominal dose (% ND, i.e., the amount of drugs placed in the reservoir of the nebulizer or contained in the puffs of the metered-dose inhaler at the beginning of experiments) or as percentage of inhaled dose (% ID, i.e., the amount of drugs that reach the distal tip of the artificial airways). The penetration of the aerosol particles into the lungs was evaluated by the penetration index. The penetration index was calculated using the outer to the inner lung-deposition region ratio normalized to the lung volume as described previously [13]. The inter-subject variability for the lung deposition data was characterized using the coefficient of variation (CV, expressed as a percentage) or the dispersion around the median.

## Results

**Fig. 1:**
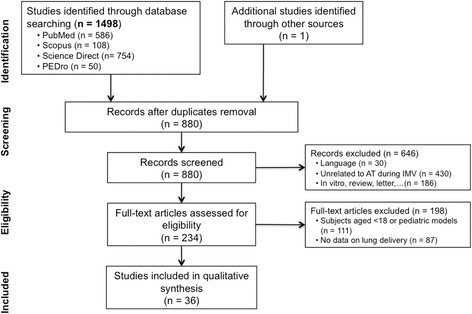
Flow diagram for study selection according to Moher et al. [11] AT aerosol therapy, IMV invasive mechanical ventilation

The flow diagram for study selection is depicted in Fig. 1. Among 234 articles assessed for eligibility, 36 studies were included and comprised 26 clinical studies (see Additional file 2: Table S1) [14–38] and 10 experimental studies (see Additional file 2: Table S2) [39–48]. The Downs and Black scores were 20 ± 2 and 18.5 ± 0.5 for the clinical and experimental studies, respectively (see Additional file 2: Table S3). Twenty clinical studies evaluated critically ill ventilated patients who were specifically suffering from documented nosocomial lung infection (ventilator-associated tracheobronchitis (VAT) or ventilator-associated pneumonia (VAP)) or were ventilated for others reasons such as chronic obstructive pulmonary disease (COPD), acute respiratory distress syndrome (ARDS) or other types of respiratory infection. Six studies were performed in ventilated patients without critical illness. These patients had lung cancer or were in the postoperative phases following cardiac, abdominal or neurological surgery. Experimental studies were performed in ventilated piglets (9 of 10 studies) or dogs (1 of 10 studies). Two thirds of the studies included in this systematic review were comparative (25 of 36 studies) (different populations, devices or administration techniques), and most were not randomized (18 of 25 studies) and were nonblinded (21 of 25 studies). Only 6 of 36 studies reported a sample size calculation, which varied from 5 to 69 patients and from 6 to 36 animals [15, 18, 20, 24, 34, 38]. Lung delivery was assessed using mass balance techniques, lung tissue sampling, imaging or pharmacokinetics techniques in 9, 9, 10 and 16 studies, respectively. Drugs of interest are detailed in Table 2.

**Table 2:** Drugs of interest

	Drugs
Antibiotics	Amikacin and amikacin sulfate [27, 28, 32, 34, 39, 40, 43, 44, 49], colistin or colistimethate sodium [14, 16, 46], ceftazidime [38, 41, 42, 48, 49], pentamidine [21], gentamycin [31, 36], tobramycin [15, 25], vancomycin [31], fosfomycin [32], imipenem [15] or teicoplanin [45]
Tracer labeled with technetium-99 m	Diethylenetriaminepentaacetic acid [18, 24, 25, 29], pertechnetate [19], sulfur colloid [19], albumin [22, 23, 35, 37] or fenoterol [20]
Bronchodilators	Albuterol [17, 30], fenoterol [20] or ipratropium bromide [26, 33]
Other	Cisplatin [47]

### Drugs reaching the distal tip of artificial airways

**Fig. 2:**
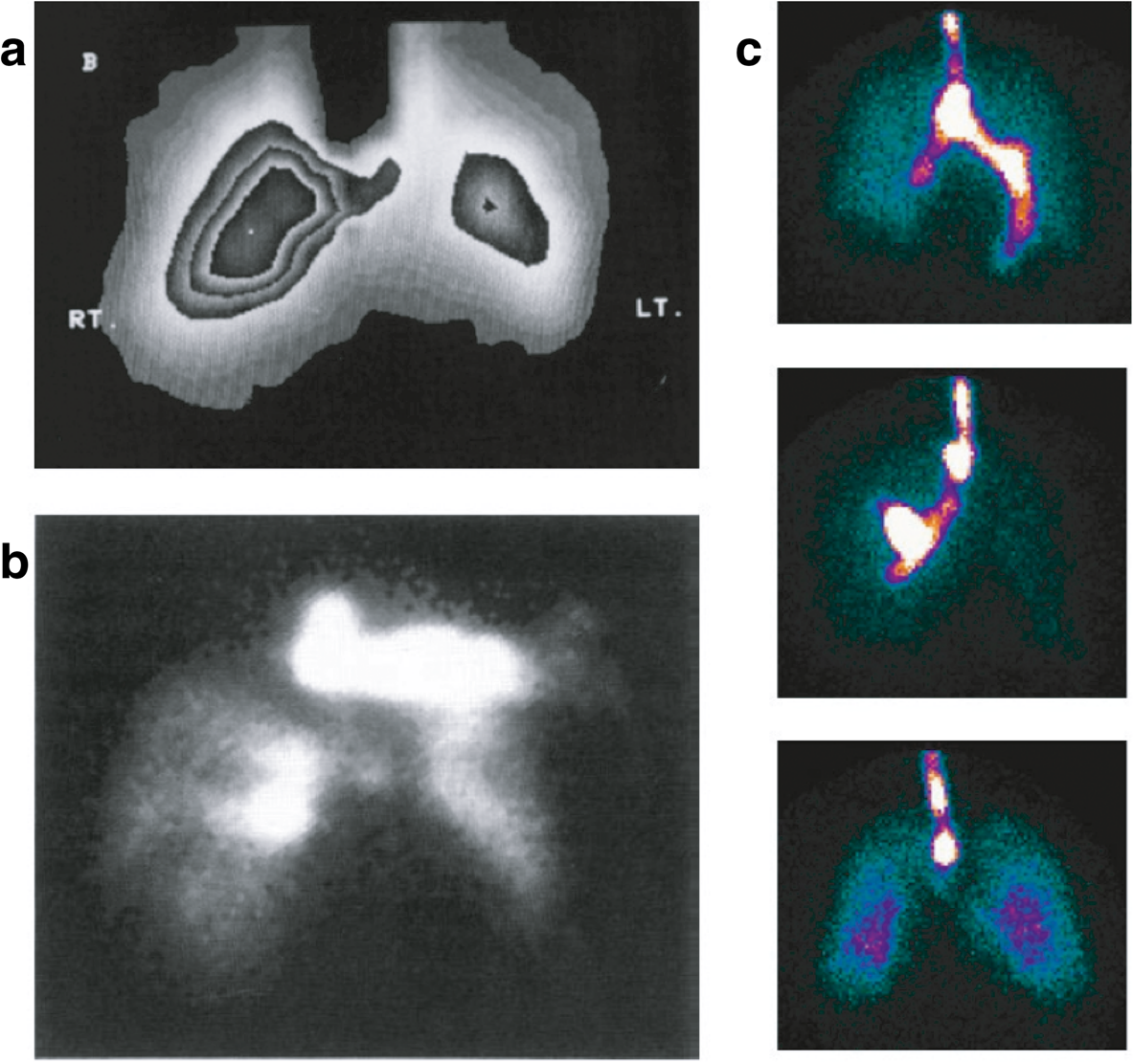
Anteroposterior acquisition using planar scintigraphy for radiolabeled aerosol deposition assessment in an intubated patient after open-heart surgery (from Thomas et al. [37], with permission) (**a**), a tracheotomised critically ill patient (from O’Riordan et al. [35], with permission) (**b**) and three intubated neurosurgical patients ventilated in volume control mode (from Dugernier et al. [18] with permission) (**c**). Even if lung outlines suggested that inhaled drugs reached the lung periphery, these images illustrate that the majority of drugs impacted proximally in the artificial airways and particularly in the trachea and large bronchi. High deposition in the endotracheal tube, the trachea and the main bronchi has been masked to improve lung definition (**a**)

Using the mass balance technique (i.e., rinsing of the ventilator circuit), authors reported 22% to 66% ND antibiotics reaching the distal tip of the tracheostomy cannula or the endotracheal tube [36, 39, 41–46, 48]. Imaging deposition studies revealed that the trachea and large bronchi represent the major site of drug deposition (Fig. 2) [18, 25, 29, 35, 37]. The study by Klockare et al. [24] reported a 49% ID of radiolabeled drug deposited in the trachea and the main bronchi of nine critically ill patients whereas 51% was distributed in both lungs with 14% in the lobar and segmental bronchi using single photon emission tomography combined with a computed tomography scanner (SPECT-CT).

### Aerosol delivery to the lungs

The highest drug doses deposited into the lungs (from the hili to the periphery, 38% ND) were obtained using a metered-dose inhaler (MDI) combined with an inhalation chamber at the Y-piece [30]. Lung doses from 3 to 7% ND and 1 to 16% ND were reported with constant-output jet nebulizers [25, 29] and inspiratory synchronized jet nebulizers [19, 22, 23, 29, 37] whereas a 5% ND was reported with a constant-output ultrasonic nebulizer [23]. Only one scintigraphic study assessed aerosol delivery in patients using a constant-output vibrating-mesh nebulizer and measured lung doses of only 10–15% ND [18].

### Aerosol distribution between both lungs

Two studies comparing aerosol delivery using a constant-output UN or direct instillation in the endotracheal tube observed a more homogeneous distribution of drugs with a nebulizer [15, 24]. Using SPECT-CT, Klockare et al. [24] measured 33% and 17% ID of radiolabeled drug in the right and the left lung after aerosol administration compared with 67% and 7% ID after instillation. A similar distribution of radiolabeled drug was measured in both lungs of patients post neurosurgery, with a trend towards a greater right to left lung-deposition ratio (from 1.39 to 3.33) [18]. Four studies enrolling critically ill or postoperative patients following open-heart surgery observed a lower left lung deposition in comparison with that of the right lung [22–24, 37].

### Aerosol penetration into the lungs

Ferrari et al. [41] reported 10% ND ceftazidime in sub-pleural specimens (i.e., homogenized bronchioles and alveoli) of healthy piglets whereas 50% ND was deposited into the trachea and proximal airways. Similar concentrations of amikacin were measured in sub-pleural specimens from both lungs, different lobes and dependent and nondependent regions from the lower lobes of healthy piglets on the first day of intubation [44]. However, a significant reduction in concentrations in dependent lung regions after prolonged mechanical ventilation was observed (50 to 400 μg/g after 24 h vs 20 to 60 μg/g after 72 h of mechanical ventilation, *p* < 0.05) [40]. Moreover, five studies of infected piglets showed a significant reduction of antibiotic deposition in subpleural specimens of lung regions with severe bronchopneumonia characterized by a massive aeration loss in comparison with partially aerated lung regions with mild bronchopneumonia [39, 42, 43, 46, 48].

Postoperative scintigraphic studies reported a penetration index (normalized O/I ratio) in patients of 0.32 to 0.75, which indicated a predominant proximal deposition [18, 37]. Eight pharmacokinetics studies reported a significant amount of antibiotics in tracheal secretions and substantial epithelial lining fluid (ELF) concentrations [14, 15, 27, 28, 31, 32, 34, 36].

### Variability in lung deposition

The intersubject variability of lung deposition ranged from 9 to 62% CV in the scintigraphic studies whatever the device used (nebulizers or metered-dose inhalers) [18–20, 22, 23, 29, 35, 37]. Dugernier et al. [18] reported a wide variability in the right to left lung-deposition ratio (3.33 (0.7–5.38) in VCV and 1.39 (0.91–2.05) in PSV). The penetration index also varied among subjects with a CV of approximately 50% [18, 37]. Pharmacokinetics studies observed from 14 to 85% CV of antibiotic concentrations in tracheobronchial secretions [31, 32, 34, 36] and from 60 to 91% CV in ELF [14, 15, 27, 28].

### Deposition in the ventilator circuit, artificial airways and nebulizer retention

**Fig. 3:**
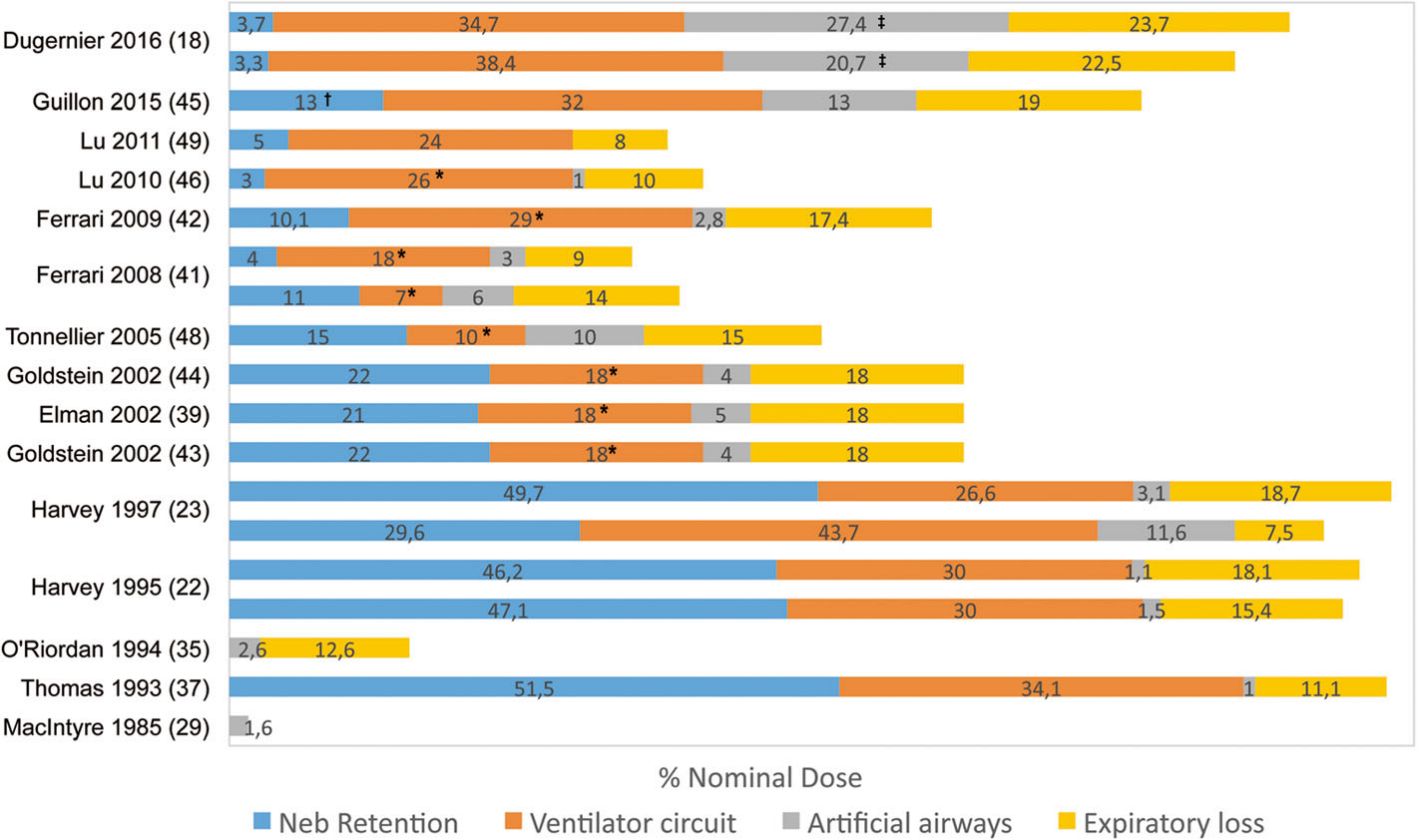
Extrapulmonary deposition expressed as percentage of nominal dose of nebulized drugs (Neb) during invasive mechanical ventilation. O’Riordan et al. [35] reported drug percentage trapped in the endotracheal tube during inspiration only (7% of nominal dose was exhaled particles trapped during expiration). This was not differentiated in other studies. *Drug deposition in the inspiratory limb only, the expiratory limb was not included. ^†^Drug retention in the nebulizer reservoir, the T-piece and the Y-piece. ^‡^Drug deposition in the endotracheal tube, the trachea and main bronchi

Fifteen studies measured drug retention within nebulizers and the circuit [18, 22, 23, 29, 35, 37, 39, 41–46, 48, 49]. No data on aerosol loss with MDIs have been described. Drug doses retained in the nebulizer reservoir and the T-piece were approximately 50% ND with jet nebulizers, 15% to 30% ND with ultrasonic nebulizers and 3% to 10% ND with vibrating-mesh nebulizers. While drugs deposited in the artificial airways and/or the trachea and the main bronchi varied from 1 to 27% ND, drugs trapped in the ventilator circuit varied from 10% to 44% ND. Drug loss during expiration was 7 to 22% ND (Fig. 3).

### Administration technique

Vibrating-mesh nebulizers are preferred over jet and ultrasonic nebulizers to deliver antibiotics (50% vs 18 and 32% of inhaled antibiotics studies, respectively). Vibrating-mesh and ultrasonic nebulizers demonstrated similar efficacy in 10 healthy piglets [41]. On one hand, greater postoperative efficacy of ultrasonic nebulizers compared to jet nebulizers was suggested in seven patients [23]. On the other hand, Lee et al. [26] did not report higher efficacy for a recent vibrating-mesh nebulizer compared to a conventional jet nebulizer. However, the authors measured 46% ND in the T-piece of the vibrating-mesh nebulizer, which questioned its efficiency in generating aerosol particles [26]. Combining a nebulizer with an inhalation chamber may significantly increase lung doses, as suggested by the scintigraphic study by Harvey et al. [22] Actuating an MDI in an inhalation chamber placed at the Y-piece allowed a 1.5-fold to 4-fold increase in lung doses of bronchodilators [20, 30].

The characteristics of mechanical ventilation during aerosol therapy are detailed in Additional file 2: Table S4. Most of the characteristics were not reported in a majority of studies. Only five clinical studies [14, 17, 18, 35, 49] reported all principal potential confounders, which was in contrast to all of the experimental studies. Lu et al. [49] standardized the administration technique to deliver inhaled antibiotics in 20 patients with VAP; a constant-output vibrating-mesh nebulizer was placed on the inspiratory limb at 10 cm of the Y-piece, specific ventilator settings were used (tidal volume of 8 mL/kg, respiratory rate of 12 c/min, duty cycle of 50%, constant and low inspiratory flow rate inferior to 30 L/min and end-inspiratory pause of 20%), and a heating-humidifier system was not used. but the heat and moisture exchanger filter was removed from the circuit during inhalation. Using this optimized technique, the authors observed 63% ND reaching the inlet of the endotracheal tube based on 37% ND extrapulmonary deposition. Among ventilation-related factors, Dugernier et al. [18] demonstrated the benefit of volume-controlled ventilation to increase lung doses in comparison with a spontaneous breathing pattern in pressure support (15.1 vs 10.5% ND, *p* < 0.05). Miller et al. [31] measured a threefold increase in tracheobronchial concentrations of antibiotics when administrating the aerosol in a dry ventilator circuit instead of a heated-humidified circuit (8.1 ± 1.5 *vs* 2.2 ± 0.4 μg/mL/mg, *p* < 0.001). Using helium instead of nitrogen in inhaled gas was found to increase ceftazidime concentrations in sub-pleural lung specimens of healthy pigs from 383 ± 84 with N_2_-O_2_ to 576 ± 141 μg/g with He-O_2_. However, the concentrations were similar in infected lung segments whichever the inhaled gas [48].

## Discussion

This article describes the first systematic review to evaluate in vivo deposition of aerosolized drugs during invasive mechanical ventilation. Lung deposition of 38% ND was reported with metered-dose inhalers which was not different from the doses reported in spontaneously breathing subjects [50]. However, lung deposition up to 16% ND was reported with nebulizers, which is likely impaired by inadequate administration techniques generating substantial aerosol loss in the ventilator circuit. Lung deposition was highly variable and mostly occurred in proximal airways, according to which type of device was used. Although the high concentrations of nebulized antibiotics measured in the ELF of infected patients (in comparison with the intravenous administration) suggested effective delivery to alveoli, the deposition in the distal lung parenchyma and different lung regions has never been quantified comprehensively in patients, especially in infected areas.

High inhaled doses (up to 66% ND) were deducted from the mass balance technique [36, 39, 41, 42, 44–46, 48]. The ND of inhaled antibiotics suggested for the treatment of VAP is based on this proportion [49]. However, lung doses may be overestimated due to limitations inherent to the mass balance technique, which primarily include incomplete rinsing of the ventilator circuit and drug mass. The drug trickle from the artificial airways into the trachea can be quantified as having been delivered to the lungs. These overestimations may be substantial especially when the nebulizer is close to the patient (see Fig. 2).

There are reports from studies of measurements of 1 − 16% ND of drugs deposited in the lungs of ventilated patients with nebulizers [18–20, 22, 23, 29, 35, 37]. While efficient bronchodilation was observed with such doses [51, 52], reaching therapeutic levels of antibiotics may be more challenging. These values are low in comparison with that reported in spontaneously breathing patients using similar models of jet or vibrating-mesh nebulizer (15 − 35% ND) [53–56]. Historically, mechanical ventilation has been considered to be a barrier to drug delivery [17, 29]. However, lung deposition is conditioned by the administration technique. An important result of this review is that most in vivo deposition studies were performed before in vitro studies that assessed the factors influencing aerosol delivery during mechanical ventilation were performed. Those in vitro studies reported substantial inhaled doses with jet nebulizers (up to 45% ND) [57, 58], ultrasonic nebulizers (up to 25% ND) [59, 60] and vibrating-mesh nebulizers (up to 72% ND) [61–64].

The following five factors potentially explain the low drug deposition: the poor efficiency of jet nebulizers [19, 22, 23, 37] (ID from 10 to 15% ND reported in vitro [58, 65]) or ultrasonic nebulizers with a voluminous reservoir [23] (ID of 15% ND reported in vitro [60]), the inadequate position in the ventilator circuit increasing either deposition on the inspiratory limb when the nebulizer was placed too far away from the patient [19] or aerosol loss in the expiratory flow when the nebulizer was placed too close [18, 22, 23, 25, 29, 37] (less than 15 cm, even for inspiratory synchronized jet nebulizers considering the delayed synchronization reported in vitro [66]), the use of a heated-humidified ventilator circuit [22, 23, 37] and the absence of standardized optimal ventilator settings. Furthermore, patient-related factors (e.g., COPD, ARDS, open-heart surgery) may also influence aerosol deposition [19, 22, 23, 29, 37]. O’Riordan et al. [35] optimized the administration technique using an inspiratory synchronized jet nebulizer, as determined in vitro [31, 57, 58] and measured higher lung doses of radiolabeled drug delivered to ventilated patients than reported in other scintigraphic studies not implementing optimized techniques (15% vs 1–3% ND) [19, 22, 23, 29, 35].

Although a homogeneous distribution between both lungs has been observed in ventilated patients with healthy lungs, there is a trend towards higher physiologic right lung deposition, as suggested in healthy volunteers [67, 68]. Scintigraphic studies in critically ill patients [24] or patients undergoing open-heart surgery [22, 23, 37] report higher right lung deposition, probably associated with impaired left lung ventilation. Scintigraphic studies report major deposition in proximal airways with penetration indexes below 1 and a greater proportion of radiolabeled drug deposited in the trachea and large bronchi [18, 24, 37].

The pathologic condition of the lung (secretion plugs or inflammatory condensation [42, 43, 46, 48], atelectasis [40], postoperative complication [22, 23, 37], or chest trauma [38]) alter aerosol distribution and penetration. As demonstrated by Elman et al. [39], the higher the aeration loss in a lung region, the lower the aerosol deposition. Aerosol penetration is also influenced by the particle size characterized by the median mass aerodynamic diameter (MMAD) [69]. However, the MMAD inferior to 3 μm measured at the distal tip of the endotracheal tube in most studies supports good distal penetration of the aerosol [18, 23]. High inspiratory flow promotes both turbulence and inertial impaction favoring particle deposition in the ventilator circuit and proximal airways, which reduces distal delivery [62]. Controlling and decreasing the inspiratory flow rate and reducing flow turbulence using lower density gases such as helium, reduces aerosol retention within the circuit in bench studies [62, 70, 71] and increases distal deposition in ventilated animals with healthy lungs [48, 72].

Aerosolized drugs may reach distal airways, as suggested by the higher antibiotic concentrations in dissected sub-pleural specimens from ventilated piglets (3-fold to 30-fold) [39, 42–44, 46] or in the ELF recovered from BAL in patients with VAP [14, 16, 38] obtained using the inhalation route instead of the intravenous administration. The Pulmonary Drug Delivery System (PDDS, Nektar Therapeutics, San Carlos, CA, USA) is a recent inspiratory synchronized vibrating-mesh nebulizer specifically designed for amikacin sulfate delivery for the treatment of VAP. Two in vitro studies have demonstrated the accurate synchronization of the PDDS with inhaled dose from 50 to 72% ND [63, 64]. Highly superior peak concentrations of amikacin were obtained in the tracheal secretions (2500-fold [34]) and the ELF (500-fold [27]) recovered from infected areas of VAP patients when compared with the intravenous administration [73, 74]. However, BAL fluid or endotracheal suctioning may both be contaminated by highly concentrated tracheobronchial secretions or particles impacted in the lumen of the artificial airways. No imaging data have been reported to confirm the better aerosol deposition with the inspiratory synchronized vibrating-mesh nebulizer compared with available nebulizers.

Clinicians should be aware of a high inter-subject variability of lung deposition in terms of lung doses, right and left lung distribution and penetration from the central airways to the lung periphery. Potential explanations include the fact that the MMAD differs between nebulizers of the same type and characterizes the deposited particle distribution through the airways [58, 75]. Moreover, patients themselves differ with respect to morphology, lung anatomy, lung pathology [76], and nonstandardized breathing patterns. However, in vitro studies demonstrated the variable inhaled doses while varying the respiratory rate, inspiratory time, inspiratory flow and the tidal volume [58, 60, 62, 65, 70]. The limitations of the deposition assessment methods may have also altered the measurements such as unstandardized lung outlines for scintigraphic analysis, BAL fluid or endotracheal suctioning sample contamination and mini-BAL measurements in different lung segments [14, 27, 32, 64].

The ventilator circuit (10 − 43% ND) and, to a lesser extent, the artificial airways, filter a substantial fraction of emitted particles, as suggested by the higher MMAD measured at the outlet of the nebulizer than the MMAD at the distal tip of the endotracheal tube [23, 44]. When the nebulizer is positioned close to the patient, artificial airways trap a significant amount of particles. A small fraction of impacted particles remains in the internal lumen of the endotracheal tube whereas the majority trickles into the trachea (and the right main bronchi), as demonstrated by the 20 − 27% ND measured in these areas by Dugernier et al. [18] This phenomenon is important, as it may lead to major overestimation of aerosol lung delivery when estimated through the mass balance technique. In contrast, scintigraphic deposition studies enable correct visualization of the site of aerosol deposition.

Most studies have focused on inhaled antibiotics, which require a rigorous administration technique with nebulizers. Several methods to improve aerosol delivery to the lungs have been emphasized in this systematic review, as demonstrated in vitro (Table 3) [6]. In their phase II trial, Lu et al. [49] optimized the administration technique using a checklist form. The authors found interesting results in 20 patients with VAP receiving inhaled amikacin and ceftazidime alone without intravenous therapy in comparison to 20 patients with VAP receiving intravenous antibiotics. Similar clinical cure and superinfection rates with other microorganisms and successful treatment of patients infected with intermediate strains in the aerosol group, suggested efficient antibiotic delivery to the infected lung site [49]. No in vivo aerosol deposition evaluation was performed in this study to link the optimized aerosol technique to improved deposition and clinical outcome.

**Table 3:** Practical recommendations to improve inhaled drug deposition with nebulizers

Using vibrating-mesh nebulizers with minimal drug retention and no risk of protein denaturation as observed with ultrasonic nebulizers [18, 27, 28, 34, 41]
Promoting inspiratory synchronized nebulizers [27, 28, 31, 35]
Combining an inhalation chamber with constant-output nebulizers (to be confirmed in further studies) [22]
Generating aerosol particles in a dry circuit^a^ [31]
Controlling the breathing pattern (high T_insp_/T_TOT_^a^, low inspiratory flow) in volume control mode [18]
Using a helium-oxygen mixture as inhaled gas [48]

^a^Probably not relevant with a recent prototype of inspiratory synchronized vibrating-mesh nebulizer, as suggested by Luyt et al. [27]

The administration technique varied greatly among all clinical studies that assessed lung deposition in vivo. Of note, the ventilator settings were not standardized and varied between patients in those studies, unlike in the study of Lu et al. [49]. Recent international surveys reported that recommendations to improve aerosol delivery are not regularly respected in current practice due to insufficient knowledge and the absence of a standardized protocol. [1, 77]. Reviewing the administration technique raised limitations to apply the current scientific knowledge by investigators. Many factors influencing lung deposition with available nebulizers are difficult to control in routine practice such as financial concerns according to the type of nebulizer (e.g., jet nebulizers for antibiotic delivery), the ventilator settings necessary for adequate ventilation, sedative infusion to adapt the patient to the ventilator (especially for prolonged synchronized nebulization or frequent administrations), heating-humidification of inhaled gases in patients with ARDS or COPD, expiratory loss due to the bias flow fixed by the manufacturer in most ventilators and high turbulent flows that induce inertial impactions in different components of the ventilator circuit, in the artificial airways and the trachea. The potential advantages of accurate synchronization (i.e., the closest position of the nebulizer to the patient) of emerging inspiratory synchronized vibrating-mesh nebulizers are several when compared with all available nebulizers: higher inhaled doses through minimal impact on the ventilator circuit and minimal expiratory loss, ventilator compatibility (ventilator settings, bias flow, components of the circuit and heated humidification) and no need to disconnect the circuit (filter or nebulized removal) and hence, lower risk of alveolar de-recruitment. These prototype inspiratory synchronized vibrating-mesh nebulizers may help to standardize an efficient, safe and feasible administration technique.

Future goals in this field include assessment of the intrapulmonary and extrapulmonary deposition to define and standardize the administration technique (i.e., whether it is necessary to adapt mechanical ventilation characteristics). Human lung deposition studies should be promoted as a “bridge” between in vitro and clinical efficacy studies [78]. While pharmacokinetics studies are limited to the assessment of whole lung deposition, scintigraphic studies may help to assess the deposition of the aerosol in different locations from the ventilator circuit to the patient. Combining a high-resolution computed tomography (CT) scan with SPECT-CT acquisitions may provide essential information on anatomical regional lung deposition (lobar analysis). However, the radioactivity exposure should be discussed [79]. Further studies are needed to test emerging devices (e.g., inspiratory synchronized nebulizers, dry powder inhalers and spacers), new drug formulations (e.g., inhalable liposome formulations or nanoparticles) and anti-infective agents (e.g., antibodies, phages). Studies aimed at developing personalized medicine may offer the possibility to confirm the ability to reach the distal airways, especially in the pathologic area (healthy vs infected lungs or focal vs diffuse infections).

This systematic review has several limitations inherent to the heterogeneity of the studies. First, comparing lung delivery rates among studies is complicated due to variable characteristics known to influence aerosol delivery: the population (human vs animal, healthy lung vs pathologic change in the lung), the aerosol device, the aerodynamic or physicochemical properties of inhaled drugs and the deposition assessment methods [6, 76]. Mechanical ventilation characteristics varied also among studies due to the evolution in the management of mechanically ventilated patients (ventilator settings and circuit) during the last 30 years and the absence of a standardized delivery technique. Second, the results from studies with small sample sizes are highly sensitive to confounding factors. The confounders were partially described and most studies did not calculate the needed sample size. The small sample size of the studies included in this review may have contributed to the variability in lung deposition observed in addition to these confounders.

## Conclusions

Aerosol delivery to mechanically ventilated patients has improved throughout the years. However, lung depositions lower than 20% ND were reported with nebulizers due to suboptimal conditions of administration that induced high aerosol loss in the ventilator circuit given that most deposition studies did not incorporate scientific knowledge subsequently gained from in vitro studies. Moreover, most studies revealed highly variable lung deposition rates in terms of doses and locations. Several factors related to the subject or mechanical ventilation that cannot be controlled probably account for this heterogeneity in part. The administration technique with nebulizers should be improved in ventilated patients with the final goal to ensure an efficient but safe, feasible and reproducible technique. Modern optimized nebulization techniques should be tested using imaging techniques to confirm the substantial distal deposition, even in infected lung areas, as reported in pharmacokinetics studies and suggested in clinical phase II studies.

## Additional files

Additional file 1: Complementary information related to the search strategy, selection criteria, data extraction and data expression. It also includes the full electronic search strategy (detailed search equation) for the Pubmed database. (DOCX 55 kb)

Additional file 2: Table S1. Characteristics and results of the clinical studies. **Table S2**. Characteristics and results of the experimental studies. **Table S3.** Downs and Black score of the included studies. **Table S4.** Mechanical ventilation characteristics during inhalation. (DOCX 209 kb)

## Abbreviations

%ID: Percentage of inhaled dose
%ND: Percentage of nominal dose
ARDS: Acute respiratory distress syndrome
BAL: Bronchoalveolar lavage
COPD: Chronic obstructive pulmonary disease
CV: Coefficient of variation
ELF: Epithelial lining fluid
ID: Inhaled dose
MDI: Metered-dose inhaler
MMAD: Mass median aerodynamic diameter
ND: Nominal dose
PDDS: Pulmonary Drug Delivery System
PSV: Pressure support ventilation
SPECT-CT: Single photon emission tomography combined with a computed tomography scanner
VAP: Ventilator-associated pneumonia
VAT: Ventilator-associated tracheobronchitis
VCV: Volume controlled ventilation
