# Supplementary material for: Aerosol delivery during invasive mechanical ventilation: a systematic review
Source: Crit Care. 2017 Oct 21;21:264. doi: 10.1186/s13054-017-1844-5 (PMC5651640; doi:10.1186/s13054-017-1844-5)
Supplement: Supplementary file 1 — Complementary information related to the search strategy, selection criteria, data extraction and data expression. It also includes the full electronic search strategy (detailed search equation) for the Pubmed database. (DOCX 55 kb) [file 13054_2017_1844_MOESM1_ESM.docx]

**Aerosol Delivery during Invasive Mechanical Ventilation:**

**A systematic Review**

Jonathan Dugernier, P.T., Stephan Ehrmann, M.D., Ph.D., M.Sc., Thierry Sottiaux, M.D., Jean Roeseler, P.T., Ph.D., Xavier Wittebole, M.D., Thierry Dugernier, M.D, Ph.D., François Jamar, M.D., Ph.D., Pierre-François Laterre, M.D., Gregory Reychler, P.T., Ph. D.

Additional file 1

**Search Strategy**

The systematic search was performed in the Pubmed, Science Direct, Scopus and PeDRO database by one investigator (JD) from 1985 to Aug 2016. Key terms were “Aerosol, Metered-dose inhaler, MDI, PMDI, Dry powder inhaled, Nebulizer, Nebulization, Nebulized particles or Inhale”, “Invasive mechanical ventilation, ventilator-associated pneumonia, critically ill or piglets” and “Deposition, Delivery, Pharmacokinetics, Scintigraphy, BAL or Blood”. Key terms as well as their variations were used as detailed in the equation in supplemental material.

**Selection Criteria**

Titles and abstract were reviewed by one investigator (JD) whereas full-articles were assessed for eligibility independently by two investigators (JD and GR). Any doubt regarding reviewed titles and abstracts were discussed and disagreement were resolved by consensus. Original research articles were included according to inclusion criteria based on participants, interventions, comparisons, outcomes and study design (PICOS) (**Table 1)**. Lung deposition was defined as the primary outcome and data on extrapulmonary deposition as a secondary outcome. When a study assessed both lung deposition and the clinical effect of the inhaled drug only the lung deposition assessment was reported in the review. Articles published in other language than English and French, reviews and meta-analysis, surveys, *in vitro* studies, pediatric studies (including subjects aged under 18 years or experimental models of pediatric/neonatal mechanical ventilation) and studies providing only clinical effect of inhaled drugs were excluded.

**Data Extraction and Study Quality Assessment**

Data extraction from eligible studies was performed in duplicate by one investigator (JD). The second extraction was blinded to the results of the previous extraction. Extracted data were checked independently by a second investigator (GR).

The Downs and Black scale defined the methodological quality of eligible studies.^1^ Two investigators (JD and GR) were involved in the risk of bias assessment. Disagreements were resolved by consensus.

Data extraction involved the study design, the population, the administered drug, the interventions i.e. the administration technique used to deliver the inhaled drug and methods used to assess aerosol delivery, and the outcomes of interest, i.e. the pulmonary deposition (lung doses, distribution and penetration) and the extrapulmonary deposition (i.e. in the ventilator circuit and the artificial airways, the expiratory loss and the device retention). The administration technique of aerosol delivery was characterized by the aerosol device (metered-dose inhaled (MDI) or jet, ultrasonic or vibrating-mesh nebulizer), its position on the ventilator circuit, the type of inspired gas, the presence of a heated-humidifier, the ventilation mode, the bias flow imposed by the ventilator and the breathing pattern (tidal volume, respiratory rate, duty cycle, peak inspiratory flow rate, inspiratory pause, positive expiratory pressure and the inspired oxygen fraction).

**Data Expression**

Data were expressed as mean ± standard deviation or median (25-75% interquartile range). Lung deposition data were expressed as percentage of nominal dose (i.e. the amount of drugs placed in the nebulizer reservoir or contained in the canister of the MDI at the beginning of experiments) or as percentage of inhaled dose (i.e. the amount of drugs that reach the distal tip of the artificial airways). Drug concentration data from lung tissue sampling were expressed as microgram of drugs per milligram of tissue (µg/mg). Lung tissue sampling was performed in lung regions with bronchopneumonia classified as mild (bronchiolitis, small foci of pneumonia, interstitial pneumonia) or severe (confluent pneumonia, necrotizing pneumonia).^2^ Drug concentrations in tracheobronchial secretions, epithelial lining fluid, plasma or urine were expressed in microgram or picogram of drugs per milliliter (µg/mL or pg/mL). The penetration of the aerosol particles into the lungs is defined by the penetration index. The penetration index is calculated using the outer to the inner lung deposition region ratio (O/I) normalized to the lung volume as described previously.^3^

The intersubject variability of lung deposition data was characterized using a coefficient of variation (CV, expressed as percentage) or the dispersion around the median.

**Search equation**

(Meter* OR MDI OR nebuli* OR "dry powder" OR aerosol* OR inhale*) AND ("mechanical ventilation" OR "invasive ventilation" OR "invasively ventilated" OR "mechanically ventilated" OR "critically ill" OR "ventilator associated pneumonia" OR piglets) AND (deposit* OR deliv* OR pharmacokinetics OR scintigraphy OR BAL OR plasma)

Search details

((meter[All Fields] OR meter'[All Fields] OR meter's[All Fields] OR meter2[All Fields] OR meter3[All Fields] OR meter4[All Fields] OR metera[All Fields] OR meterage[All Fields] OR meterages[All Fields] OR meteralpha[All Fields] OR meteraminol[All Fields] OR meteran[All Fields] OR meterangelis[All Fields] OR meterani[All Fields] OR meteras[All Fields] OR meterazina[All Fields] OR meterazine[All Fields] OR meterbuen[All Fields] OR metercercariae[All Fields] OR meterdez[All Fields] OR meterdomini[All Fields] OR metere[All Fields] OR metere1[All Fields] OR metereau[All Fields] OR metered[All Fields] OR metered'[All Fields] OR metereddose[All Fields] OR meterei[All Fields] OR meterelin[All Fields] OR metereline[All Fields] OR meterelliyoz[All Fields] OR metereo[All Fields] OR metereologia[All Fields] OR metereologic[All Fields] OR metereological[All Fields] OR metereologicas[All Fields] OR metereologiche[All Fields] OR metereologici[All Fields] OR metereologicos[All Fields] OR metereologisch[All Fields] OR metereology[All Fields] OR metereopathology[All Fields] OR metereopatologia[All Fields] OR metereorological[All Fields] OR metereotropowe[All Fields] OR meteresadamiani[All Fields] OR meterev[All Fields] OR meterf[All Fields] OR metergasis[All Fields] OR metergic[All Fields] OR metergin[All Fields] OR metergina[All Fields] OR meterginem[All Fields] OR meterginy[All Fields] OR metergolin[All Fields] OR metergolina[All Fields] OR metergoline[All Fields] OR metergoline's[All Fields] OR meteria[All Fields] OR meterial[All Fields] OR meteriala[All Fields] OR meterialam[All Fields] OR meteriale[All Fields] OR meterials[All Fields] OR meterialy[All Fields] OR meterice[All Fields] OR meterie[All Fields] OR metering[All Fields] OR meteriological[All Fields] OR meteris[All Fields] OR meterissian[All Fields] OR meterko[All Fields] OR meterkom[All Fields] OR metermal[All Fields] OR metern[All Fields] OR meterna[All Fields] OR meternal[All Fields] OR meternicovej[All Fields] OR meternita[All Fields] OR meterno[All Fields] OR meteroaryl[All Fields] OR meterogeneity[All Fields] OR meterogline[All Fields] OR meteroglu[All Fields] OR meterogoline[All Fields] OR meteroism[All Fields] OR meteroite[All Fields] OR meterologia[All Fields] OR meterologic[All Fields] OR meterological[All Fields] OR meterologiche[All Fields] OR meterologicheskikh[All Fields] OR meterologichni[All Fields] OR meterologichnite[All Fields] OR meterologici[All Fields] OR meterologickych[All Fields] OR meterologicos[All Fields] OR meterologie[All Fields] OR meterologiques[All Fields] OR meterologisch[All Fields] OR meterologische[All Fields] OR meterologischen[All Fields] OR meterologischer[All Fields] OR meterologisches[All Fields] OR meterologists[All Fields] OR meterologo[All Fields] OR meterology[All Fields] OR meteromimetic[All Fields] OR meteropathology[All Fields] OR meteropathy[All Fields] OR meteropolitan[All Fields] OR meteroprolol[All Fields] OR meteror[All Fields] OR meterorism[All Fields] OR meterorismus[All Fields] OR meterorite[All Fields] OR meteroroligical[All Fields] OR meterorologic[All Fields] OR meterorological[All Fields] OR meterorologicheskikh[All Fields] OR meterorologicos[All Fields] OR meterorologicznych[All Fields] OR meteroroloskih[All Fields] OR meteroropathologia[All Fields] OR meterorotropie[All Fields] OR meterortropismus[All Fields] OR meterosensitivitat[All Fields] OR meterosideros[All Fields] OR meterotropism[All Fields] OR meterovi[All Fields] OR meterph905[All Fields] OR meterplan[All Fields] OR meterreau[All Fields] OR meters[All Fields] OR meters'[All Fields] OR meters2[All Fields] OR meterset[All Fields] OR metersfor[All Fields] OR meterskii[All Fields] OR metersky[All Fields] OR meterstein[All Fields] OR meterstick[All Fields] OR meterstitial[All Fields] OR meterstone[All Fields] OR metertech[All Fields] OR meterveli[All Fields] OR metervisus[All Fields] OR meterwaves[All Fields] OR meterwellen[All Fields] OR meterwellenstrahlung[All Fields]) OR MDI[All Fields] OR (nebuli[All Fields] OR nebuliae[All Fields] OR nebuliatorov[All Fields] OR nebulic[All Fields] OR nebulicola[All Fields] OR nebulied[All Fields] OR nebulier[All Fields] OR nebuliers[All Fields] OR nebulifer[All Fields] OR nebulifera[All Fields] OR nebulin[All Fields] OR nebulin's[All Fields] OR nebulins[All Fields] OR nebulisable[All Fields] OR nebulisada[All Fields] OR nebulisat[All Fields] OR nebulisate[All Fields] OR nebulisateur[All Fields] OR nebulisation[All Fields] OR nebulisations[All Fields] OR nebulisator[All Fields] OR nebulisats[All Fields] OR nebulise[All Fields] OR nebulised[All Fields] OR nebulisee[All Fields] OR nebuliser[All Fields] OR nebuliseringsterapi[All Fields] OR nebulisers[All Fields] OR nebuliseru[All Fields] OR nebulises[All Fields] OR nebuliseur[All Fields] OR nebuliseurs[All Fields] OR nebulising[All Fields] OR nebulium[All Fields] OR nebulizable[All Fields] OR nebulizables[All Fields] OR nebulizacao[All Fields] OR nebulizaci[All Fields] OR nebulizacion[All Fields] OR nebulizaciones[All Fields] OR nebulizaciou[All Fields] OR nebulizacji[All Fields] OR nebulizacoes[All Fields] OR nebulizada[All Fields] OR nebulizadas[All Fields] OR nebulizado[All Fields] OR nebulizador[All Fields] OR nebulizadores[All Fields] OR nebulizados[All Fields] OR nebulizalion[All Fields] OR nebulizant[All Fields] OR nebulizar[All Fields] OR nebulizare[All Fields] OR nebulizate[All Fields] OR nebulizated[All Fields] OR nebulization[All Fields] OR nebulizationde[All Fields] OR nebulizations[All Fields] OR nebulizator[All Fields] OR nebulizatora[All Fields] OR nebulizators[All Fields] OR nebulize[All Fields] OR nebulized[All Fields] OR nebulizer[All Fields] OR nebulizer'[All Fields] OR nebulizer's[All Fields] OR nebulizerle[All Fields] OR nebulizers[All Fields] OR nebulizers'[All Fields] OR nebulizes[All Fields] OR nebulizied[All Fields] OR nebulizing[All Fields] OR nebulizirovannogo[All Fields] OR nebulizowanej[All Fields] OR nebulizzati[All Fields] OR nebulizzatore[All Fields] OR nebulizzatori[All Fields] OR nebulizzazione[All Fields] OR nebulizzazioni[All Fields]) OR "dry powder"[All Fields] OR (aerosol[All Fields] OR aerosol'[All Fields] OR aerosol'noi[All Fields] OR aerosol's[All Fields] OR aerosol0[All Fields] OR aerosola[All Fields] OR aerosolami[All Fields] OR aerosolanwendung[All Fields] OR aerosolapplikation[All Fields] OR aerosolation[All Fields] OR aerosolausbreitung[All Fields] OR aerosolban[All Fields] OR aerosolbarne[All Fields] OR aerosolbehandeling[All Fields] OR aerosolbehandling[All Fields] OR aerosolbehandlun[All Fields] OR aerosolbehandlung[All Fields] OR aerosolbestandteilen[All Fields] OR aerosolbolusdispersion[All Fields] OR aerosolborne[All Fields] OR aerosoldeposition[All Fields] OR aerosoldepositionsmuster[All Fields] OR aerosoldesinfektion[All Fields] OR aerosoldiagnostiek[All Fields] OR aerosoldiagnostik[All Fields] OR aerosoldichte[All Fields] OR aerosoldos[All Fields] OR aerosoldosierung[All Fields] OR aerosoldruckpackungen[All Fields] OR aerosole[All Fields] OR aerosoled[All Fields] OR aerosolegerate[All Fields] OR aerosolei[All Fields] OR aerosolem[All Fields] OR aerosolen[All Fields] OR aerosoler[All Fields] OR aerosolerzeugung[All Fields] OR aerosoles[All Fields] OR aerosolform[All Fields] OR aerosolforsch[All Fields] OR aerosolforschung[All Fields] OR aerosolgabe[All Fields] OR aerosolgebundenem[All Fields] OR aerosolgebundener[All Fields] OR aerosolgenerator[All Fields] OR aerosolgeneratoren[All Fields] OR aerosolgerat[All Fields] OR aerosolgeraten[All Fields] OR aerosolgerates[All Fields] OR aerosoli[All Fields] OR aerosolia[All Fields] OR aerosoliariia[All Fields] OR aerosolic[All Fields] OR aerosolica[All Fields] OR aerosolically[All Fields] OR aerosolicas[All Fields] OR aerosolico[All Fields] OR aerosolierter[All Fields] OR aerosoliertes[All Fields] OR aerosolierung[All Fields] OR aerosolii[All Fields] OR aerosolilor[All Fields] OR aerosolima[All Fields] OR aerosolimmunisierung[All Fields] OR aerosolimpfung[All Fields] OR aerosolin[All Fields] OR aerosolinalation[All Fields] OR aerosoling[All Fields] OR aerosolinhalation[All Fields] OR aerosolinhalationen[All Fields] OR aerosolinhalations[All Fields] OR aerosolinhalationstherapie[All Fields] OR aerosolinhalationstoxizitat[All Fields] OR aerosolique[All Fields] OR aerosolisable[All Fields] OR aerosolisation[All Fields] OR aerosolisations[All Fields] OR aerosolise[All Fields] OR aerosolised[All Fields] OR aerosolised'[All Fields] OR aerosolisees[All Fields] OR aerosoliser[All Fields] OR aerosoliseret[All Fields] OR aerosoliseur[All Fields] OR aerosolisiertem[All Fields] OR aerosolisiertes[All Fields] OR aerosolising[All Fields] OR aerosolizability[All Fields] OR aerosolizable[All Fields] OR aerosolizaciones[All Fields] OR aerosolizada[All Fields] OR aerosolizados[All Fields] OR aerosolization[All Fields] OR aerosolizations[All Fields] OR aerosolizaton[All Fields] OR aerosolize[All Fields] OR aerosolized[All Fields] OR aerosolizer[All Fields] OR aerosolizers[All Fields] OR aerosolizes[All Fields] OR aerosolizing[All Fields] OR aerosolizzata[All Fields] OR aerosolizzati[All Fields] OR aerosolizzatta[All Fields] OR aerosolizzazione[All Fields] OR aerosolizzazioni[All Fields] OR aerosolkonzentration[All Fields] OR aerosollal[All Fields] OR aerosolmedizin[All Fields] OR aerosolmorphometrie[All Fields] OR aerosolnog[All Fields] OR aerosoloch[All Fields] OR aerosologia[All Fields] OR aerosologie[All Fields] OR aerosologischer[All Fields] OR aerosology[All Fields] OR aerosolom[All Fields] OR aerosolot[All Fields] OR aerosoloterapia[All Fields] OR aerosoloterapii[All Fields] OR aerosolotherapie[All Fields] OR aerosolotherapy[All Fields] OR aerosolov[All Fields] OR aerosolov'ych[All Fields] OR aerosolov'ymi[All Fields] OR aerosolova[All Fields] OR aerosolove[All Fields] OR aerosolovy[All Fields] OR aerosolovych[All Fields] OR aerosolovym[All Fields] OR aerosolovymi[All Fields] OR aerosolowej[All Fields] OR aerosolowym[All Fields] OR aerosolparticle[All Fields] OR aerosolpartikeln[All Fields] OR aerosolphysics[All Fields] OR aerosolpreparater[All Fields] OR aerosolprobleme[All Fields] OR aerosolresearch[All Fields] OR aerosols[All Fields] OR aerosols'[All Fields] OR aerosolsampling[All Fields] OR aerosolsfor[All Fields] OR aerosolspray[All Fields] OR aerosolsprays[All Fields] OR aerosolstorming[All Fields] OR aerosolteilchen[All Fields] OR aerosolterapia[All Fields] OR aerosolterapico[All Fields] OR aerosolthearpy[All Fields] OR aerosoltherapeutical[All Fields] OR aerosoltheraphie[All Fields] OR aerosoltherapie[All Fields] OR aerosoltherapy[All Fields] OR aerosolu[All Fields] OR aerosoluciones[All Fields] OR aerosolum[All Fields] OR aerosoluy[All Fields] OR aerosolvakzinierung[All Fields] OR aerosolve[All Fields] OR aerosolverfahren[All Fields] OR aerosolverteilungsmuster[All Fields] OR aerosolvolume[All Fields] OR aerosolwirkungen[All Fields] OR aerosoly[All Fields] OR aerosolyser[All Fields] OR aerosolyzed[All Fields]) OR (inhale[All Fields] OR inhaleable[All Fields] OR inhaled[All Fields] OR inhaled'[All Fields] OR inhaledsteroids[All Fields] OR inhalee[All Fields] OR inhalees[All Fields] OR inhaleexhale[All Fields] OR inhalent[All Fields] OR inhaler[All Fields] OR inhaler'[All Fields] OR inhaler's[All Fields] OR inhalerad[All Fields] OR inhalerat[All Fields] OR inhalerede[All Fields] OR inhaleren[All Fields] OR inhaleret[All Fields] OR inhalering[All Fields] OR inhalerle[All Fields] OR inhalern[All Fields] OR inhaleror[All Fields] OR inhalerr[All Fields] OR inhalers[All Fields] OR inhalers'[All Fields] OR inhalers2[All Fields] OR inhalersthat[All Fields] OR inhalerte[All Fields] OR inhalertrade[All Fields] OR inhales[All Fields] OR inhales'[All Fields] OR inhalet[All Fields] OR inhaletas[All Fields] OR inhaleted[All Fields] OR inhalets[All Fields] OR inhalets'[All Fields] OR inhaletten[All Fields] OR inhalex[All Fields] OR inhalexpert[All Fields])) AND ("mechanical ventilation"[All Fields] OR "invasive ventilation"[All Fields] OR "invasively ventilated"[All Fields] OR "mechanically ventilated"[All Fields] OR "critically ill"[All Fields] OR "ventilator associated pneumonia"[All Fields] OR piglets[All Fields]) AND ((deposit[All Fields] OR deposit'[All Fields] OR deposit''[All Fields] OR deposit's[All Fields] OR deposit3d[All Fields] OR deposita[All Fields] OR depositable[All Fields] OR depositada[All Fields] OR depositadas[All Fields] OR depositado[All Fields] OR depositados[All Fields] OR depositaire[All Fields] OR deposital[All Fields] OR depositant[All Fields] OR depositarie[All Fields] OR depositaron[All Fields] OR depositary[All Fields] OR depositary's[All Fields] OR depositate[All Fields] OR depositd[All Fields] OR deposite[All Fields] OR deposited[All Fields] OR deposited'[All Fields] OR depositedsinw[All Fields] OR deposites[All Fields] OR depositi[All Fields] OR depositidn[All Fields] OR depositied[All Fields] OR depositifs[All Fields] OR depositiion[All Fields] OR depositing[All Fields] OR depositing1[All Fields] OR deposition[All Fields] OR deposition'[All Fields] OR depositional[All Fields] OR depositionally[All Fields] OR depositionand[All Fields] OR depositionby[All Fields] OR depositionclearance[All Fields] OR depositiondatasets[All Fields] OR depositioned[All Fields] OR depositioning[All Fields] OR depositionn[All Fields] OR depositionof[All Fields] OR depositionogenic[All Fields] OR depositionon[All Fields] OR depositionreductions[All Fields] OR depositions[All Fields] OR depositionscharakteristika[All Fields] OR depositionseffizienz[All Fields] OR depositionsmodell[All Fields] OR depositionsnachweis[All Fields] OR depositionson[All Fields] OR depositiontrade[All Fields] OR depositis[All Fields] OR depositive[All Fields] OR depositivity[All Fields] OR depositivores[All Fields] OR depositlike[All Fields] OR deposito[All Fields] OR depositon[All Fields] OR depositor[All Fields] OR depositor's[All Fields] OR depositories[All Fields] OR depositors[All Fields] OR depositors'[All Fields] OR depository[All Fields] OR depositos[All Fields] OR deposits[All Fields] OR deposits'[All Fields] OR depositsare[All Fields] OR depositsdifferent[All Fields] OR depositted[All Fields] OR deposittions[All Fields] OR depositum[All Fields] OR depositwas[All Fields] OR deposity[All Fields]) OR (deliv[All Fields] OR deliva[All Fields] OR delivand[All Fields] OR delivani[All Fields] OR delivanis[All Fields] OR delivannides[All Fields] OR delivanoglou[All Fields] OR delivanova[All Fields] OR delivaries[All Fields] OR delivary[All Fields] OR delivative[All Fields] OR delivatized[All Fields] OR delive[All Fields] OR delived[All Fields] OR delivelioti[All Fields] OR deliveliotis[All Fields] OR deliveliotou[All Fields] OR deliver[All Fields] OR deliver'[All Fields] OR deliver'in[All Fields] OR deliver1982[All Fields] OR delivera[All Fields] OR deliverability[All Fields] OR deliverability'[All Fields] OR deliverable[All Fields] OR deliverable'[All Fields] OR deliverables[All Fields] OR deliverables'[All Fields] OR deliverance[All Fields] OR deliverance'[All Fields] OR deliverances[All Fields] OR deliverate[All Fields] OR deliverately[All Fields] OR deliveration[All Fields] OR deliverations[All Fields] OR delivercdnaorrnai[All Fields] OR deliverd[All Fields] OR delivere[All Fields] OR delivered[All Fields] OR delivered'[All Fields] OR delivereda[All Fields] OR deliveredby[All Fields] OR deliveredfrom[All Fields] OR deliverence[All Fields] OR deliverer[All Fields] OR deliverer's[All Fields] OR deliverers[All Fields] OR deliverers'[All Fields] OR deliverery[All Fields] OR deliveres[All Fields] OR deliverey[All Fields] OR deliverie[All Fields] OR deliveried[All Fields] OR deliverieo[All Fields] OR deliveries[All Fields] OR deliveries'[All Fields] OR deliverin[All Fields] OR delivering[All Fields] OR delivering'[All Fields] OR deliveris[All Fields] OR delivers[All Fields] OR delivers'[All Fields] OR deliverska[All Fields] OR deliverski[All Fields] OR delivert[All Fields] OR deliverthe[All Fields] OR deliverv[All Fields] OR delivervirulence[All Fields] OR delivery[All Fields] OR delivery'[All Fields] OR delivery's[All Fields] OR delivery5[All Fields] OR delivery84[All Fields] OR deliveryabout[All Fields] OR deliveryand[All Fields] OR deliverybrigham[All Fields] OR deliverycan[All Fields] OR deliverydelivery[All Fields] OR deliverydosage[All Fields] OR deliveryformatforpresentations[All Fields] OR deliveryfort[All Fields] OR deliverying[All Fields] OR deliveryman[All Fields] OR deliverymen[All Fields] OR deliveryroom[All Fields] OR deliverys[All Fields] OR deliverysystem[All Fields] OR deliveryvia[All Fields] OR deliverywas[All Fields] OR deliverywere[All Fields] OR deliverywithin[All Fields] OR delives[All Fields] OR delivet[All Fields] OR delivety[All Fields] OR delivey[All Fields] OR delivichev[All Fields] OR delivieries[All Fields] OR delivopo[All Fields] OR delivopoulos[All Fields] OR delivoria[All Fields] OR delivorias[All Fields] OR delivr'ee[All Fields] OR delivramento[All Fields] OR delivrance[All Fields] OR delivrances[All Fields] OR delivrant[All Fields] OR delivre[All Fields] OR delivred[All Fields] OR delivree[All Fields] OR delivrees[All Fields] OR delivrer[All Fields] OR delivrery[All Fields] OR delivres[All Fields] OR delivrey[All Fields] OR delivron[All Fields] OR delivry[All Fields] OR delivs1[All Fields] OR delivs2[All Fields] OR delivuk[All Fields]) OR ("pharmacokinetics"[Subheading] OR "pharmacokinetics"[All Fields] OR "pharmacokinetics"[MeSH Terms]) OR ("radionuclide imaging"[MeSH Terms] OR ("radionuclide"[All Fields] AND "imaging"[All Fields]) OR "radionuclide imaging"[All Fields] OR "scintigraphy"[All Fields]) OR BAL[All Fields] OR ("plasma"[MeSH Terms] OR "plasma"[All Fields]))

References Text 1

1. Downs SH, Black N. The feasibility of creating a checklist for the assessment of the methodological quality both of randomised and non-randomised studies of health care interventions. *J Epidemiol Community Health.* 1998;52(6):377-84.

2. Elman M, Goldstein I, Marquette CH, Wallet F, Lenaour G, Rouby JJ, et al. Influence of lung aeration on pulmonary concentrations of nebulized and intravenous amikacin in ventilated piglets with severe bronchopneumonia. *Anesthesiology.* 2002;97(1):199-206.

3. Newman S, Bennett WD, Biddiscombe M, Devadason SG, Dolovich MB, Fleming J, et al. Standardization of techniques for using planar (2D) imaging for aerosol deposition assessment of orally inhaled products. *J Aerosol Med Pulm Drug Deliv.* 2012;25 Suppl 1:S10-28.

Corresponding author

Jonathan Dugernier, P.T., M.Sc.

Soins Intensifs, Cliniques universitaires Saint-Luc, Avenue Hippocrate 10, 1200 Brussels, Belgium

[Jonathan.dugernier@uclouvain.be](mailto:Jonathan.dugernier@uclouvain.be)

Tel: 0032/ 2 764 2711

Fax: 0032/2 764 8928
