# Supplementary material for: Aerosol delivery during invasive mechanical ventilation: a systematic review
Source: Crit Care. 2017 Oct 21;21:264. doi: 10.1186/s13054-017-1844-5 (PMC5651640; doi:10.1186/s13054-017-1844-5)
Supplement: Supplementary file 2 — Characteristics and results of the clinical studies. Table S2. Characteristics and results of the experimental studies. Table S3. Downs and Black score of the included studies. Table S4. Mechanical ventilation characteristics during inhalation. (DOCX 209 kb) [file 13054_2017_1844_MOESM2_ESM.docx]

**Aerosol Delivery during Invasive Mechanical Ventilation:**

**A systematic Review**

Jonathan Dugernier, P.T., M.Sc., Thierry Sottiaux, M.D., Jean Roeseler, P.T., Ph.D., Thierry Dugernier, M.D, Ph.D., François Jamar, M.D., Ph.D., Stephan Ehrmann, M.D., Ph.D., Pierre-François Laterre, M.D., Gregory Reychler, P.T., Ph. D.

Additional file 2

Table S1, S2, S3, S4

**Table S1 (1/8)**. Characteristics and results of the clinical studies

| **Study (Year) (Ref)** | **Design** | **Population, artificial airways (ETT/TC)** | **Drug(s) or tracer** | **Administration modality (position)** | **Lung deposition analysis** | **Outcomes of interest** | **D&B score (/28)** |
| --- | --- | --- | --- | --- | --- | --- | --- |
| MacIntyre et al. (1985) [1] | Comparative | 7 ETT patients with VD *vs* 3 SB subjects | 99mTc-DTPA | C-O JN (connected to the ETT) | γ-Scinti | WLD: 2.9 ± 0.7 *vs* 11.9 ± 2.2% ND * | 19 |
| Girard et al. (1989) [2] | Comparative | 26 AIDS patients with pneumocystis pneumonia  (8 ETT *vs* 18 SB) | Pentamidine | C-O UN (Connected to the ventilator) *vs* C-O UN (via mouthpiece or facemask during CPAP) | Plasma PK | Peak concentration: 0.215 ± 0.05 *vs* 0.065 ± 0.01 µg/mL^†^ | 16 |
| Fuller et al. (1990) [3] | Randomized Comparative | 16 ETT patients with VD (7 *vs* 9) | 99mTcO_4_ (MDI) and 99mTc-SC (JN) | MDI + Spacer (20 cm of the ETT) *vs* I-synch JN (70 cm of the ETT) | γ-Scinti | WLD: 5.6 ± 1.1 *vs* 1.2 ± 0.3% ND* | 19 |

**Table S1 (2/8).** Characteristics and results of the clinical studies (Continuation)

| **Study (Year) (Ref)** | **Design** | **Population, artificial airways (ETT/TC)** | **Drug(s) or tracer** | **Administration modality (position)** | **Lung deposition analysis** | **Outcomes of interest** | **D&B score (/28)** |
| --- | --- | --- | --- | --- | --- | --- | --- |
| Le Conte et al. (1993) [4] | Comparative | (A) 5 ETT with lung cancer  (B) 5 ETT (healthy lungs) *vs* (C) 5 SB subjects | (A) Tobramycin (300 mg)  (B) and (C) Tobramycin (300 mg) + 99mTc-DTPA | C-O JN (Between the ETT and the reservoir bag) | (A) LTS in healthy lung  (B) and (C) Urinary PK and γ-Scinti (observational) | (A) Peak concentrations in 4 patients: 5.6 ± 5.5µg/g, Not detectable in 1 patient  (B) *vs* (C): Similar urinary availability: 6.6 ± 4.3 *vs* 4.3 ± 1.9% ND | 17 |
| Thomas et al. (1993) [5] | Cohort | 9 ETT patients after open-heart surgery | 99mTc-HSA | I-synch JN (Connected to the ETT) | γ-Scinti (planar) | - WLD: 2.3 ± 0.8% ND - RL and LLD: 1.5 ± 0.75 and 0.75 ± 0.3% ND - O/I: 0.54 ± 0.19 - U/L: 1.40 ± 0.58 | 19 |
| O’Riordan et al. (1994) [6] | Cohort | 7 TC patients with VD | 99mTc-HSA | I-synch JN (30 cm of the Y-piece) | γ-Scinti (planar) | Both lungs: 15.3 ± 9.5% ND | 19 |

**Table S1 (3/8).** Characteristics and results of the clinical studies (Continuation)

| **Study (Year) (Ref)** | **Design** | **Population, artificial airways (ETT/TC)** | **Drug(s) or tracer** | **Administration modality (position)** | **Lung deposition analysis** | **Outcomes of interest** | **D&B score (/28)** |
| --- | --- | --- | --- | --- | --- | --- | --- |
| Fuller et al. (1994) [7] | Randomized Comparative | 48 ETT patients with VD ((A) 11 *vs* (B) 8 *vs* (C) 18 *vs* (D) 11) | 99mTc-Fenoterol | (A) MDI (Connected to the ETT) *vs* (B) MDI alone *vs* (C) MDI + Small Spacer *vs* (D) MDI + Large Spacer (Y-piece) | γ-Scinti (planar) | WLD: (A) 3.9 ± 1.2 *vs* (B) 1.7 ± 0.7 or (C) 5.5 ± 0.5 or (D) 6.3 ± 0.6% ND (not corrected for tissue attenuation)  With (C) *vs* (B), (D) *vs* (B) ^†^ | 20 |
| Harvey et al. (1995) [8] | Comparative Cross-over | 10 ETT patients after open-heart surgery | 99mTc-HSA | I-synch JN alone *vs* I-synch JN + Spacer (15 cm of the Y-piece) | γ-Scinti (planar) | - WLD: 2.2 ± 0.7 *vs* 3.0 ± 0.8% ND^†^ - RLD: 1.5 ± 0.6 *vs* 2 ±à 0.7% ND^†^ - LLD: 0.7 ± 0.3 *vs* 0.9 ± 0.3% ND^†^ | 18 |
| Duarte et al. (1996) [9] | Comparative | 10 ETT patients with VD *vs* 10 SB volunteers | Albuterol (1 mg *vs* 0.6 mg) | MDI + Spacer (15 cm of the ETT) | Plasma PK | Peak concentration: 16.8 ± 1.4 *vs* 23.4 ± 1.9 ng/mL/puff x min^†^ | 20 |
| Harvey et al. (1997) [10] | Comparative  Cross-over | 7 ETT patients after open-heart surgery | 99mTc-HSA | I-Synch JN (15 cm of the Y-piece) *vs* C-O UN (35 cm of the Y-piece) | γ-Scinti (planar) | - WLD :2.3 ± 0.9 *vs* 5.3 ± 1.4% ND^†^ - RLD: 1.4 ± 0.5 *vs* 3.8 ± 1.6% of ND^†^ - LLD: 0.9 ± 0.5 *vs* 1.5 ± 0.8% of ND^†^ | 18 |

**Table S1 (4/8).** Characteristics and results of the clinical studies (Continuation)

| **Study (Year) (Ref)** | **Design** | **Population, artificial airways (ETT/TC)** | **Drug(s) or tracer** | **Administration modality (position)** | **Lung deposition analysis** | **Outcomes of interest** | **D&B score (/28)** |
| --- | --- | --- | --- | --- | --- | --- | --- |
| Palmer et al. (1998) [11] | Cohort | 6 TC patients with VAT and VD | 99mTc-Gentamicin (80 mg) or 99mTc-AMK | Insp synch JN (30 cm of the Y-piece) | - ETA - WR of the ventilator circuit and the TC | - Gentamicin concentration in sputum: 16.6 ± 8.1 µg/mL/mg (i.e. 1200 µg/mL for 80 mg) - ID: 21.9 ± 7.1% ND of antibiotics | 20 |
| Marik et al. (1999) [12] | RandomizedComparative | 30 ETT COPD patients (10 *vs* 10 *vs* 10) | Albuterol with MDI (450 µg) or with JN (2.5 mg) | MDI + Spacer (At the Y-piece) *vs* MDI alone (Connected to the ETT) *vs* I-Synch JN (30 cm of the Y-piece) | Urine PK (6h) | Amount recovered in urine: 169 ± 129 (38%) *vs* 409 ± 515 (9%) *vs* 41 ± 61 µg (16% ND)  ^†^ between groups | 20 |
| Wood et al. (2002) [13] | Cohort | 20 ETT trauma patients with high risk for VAP and VD | Ceftazidime (250 mg/12h) | I-synch JN (15 cm of the Y-piece) | BAL in the more severely injured lung | - Through concentration (7h after admin): 56 (2.1 – 443.9) µg/mL - Undetectable in 3 patients | 21 |

**Table S1 (5/8).** Characteristics and results of the clinical studies (Continuation)

| **Study (Year) (Ref)** | **Design** | **Population, artificial airways (ETT/TC)** | **Drug(s) or tracer** | **Administration modality (position)** | **Lung deposition analysis** | **Outcomes of interest** | **D&B score (/28)** |
| --- | --- | --- | --- | --- | --- | --- | --- |
| Miller et al. (2003) [14] | Comparative cross-over | 6 ETT patients with VD | AMK (400 mg), genta (80 mg), vanco (120 mg) | C-O *vs* insp synch JN (30 cm of the Y-piece) | ETA | - Antibiotics concentrations in secretions (nonhumidified condition): 1.8 ±0.3 *vs* 12.6 ± 1.8 µg/mL/mg neb charge* - 3-fold decrease of concentrations with humidified gas^†^ | 19 |
| Badia et al. (2004) [15] | Randomized comparative | 12 ETT patients with VD (6 *vs* 6) | Tobramycin (200 mg)  Imipenem (1000 mg) | C-O UN (At the Y-piece) *vs* ETT Instillation | 2 Bronchoscopy and 3 ETA | - Similar concentrations of tobramycin: 102 ± 61 µg/mL *vs* 142 ± 125 µg/mL - Higher concentration of imipenem due to the precipitation in the UN reservoir | 23 |
| Klockare et al. (2006) [16] | Comparative  Cross-over | 9 ETT patients with VD | 99mTc-DTPA | C-O UN (connected to the ventilator) *vs* ETT instillation | γ-Scinti (SPECT-CT) | - Similar lung deposition fraction: 51.6 *vs* 75.9 % ID - RLD: 33.2 *vs* 66.7% ID^†^ - Similar LLD: 17.4 *vs* 9.2% ID | 19 |
| Moraine et al. (2009) [17] | Randomized comparative | 38 ETT patients with VD | Ipratropium bromide (500 µg) | I-synch UN (before the humidifier *vs* At the Y-piece) | Urinary PK | Similar concentration: 0.013 ± 0.002 *vs* 0.015± 0.003 µg/mL (around 13% ND) | 20 |

**Table S1 (6/8).** Characteristics and results of the clinical studies (Continuation)

| **Study (Year) (Ref)** | **Design** | **Population, artificial airways (ETT/TC)** | **Drug(s) or tracer** | **Administration modality (position)** | **Lung deposition analysis** | **Outcomes of interest** | **D&B score (/28)** |
| --- | --- | --- | --- | --- | --- | --- | --- |
| Luyt et al. (2009) [18] | Cohort | 28 ETT/TC VAP patients with VD | AMK sulfate (400 mg/12h) | I-synch VN (connected to the ETT) | BAL in the infection-involved zone | Peak concentration on day 3: 976 (410-2,563) µg/mL | 19 |
| Luyt et al. (2011) [19] | Cohort | 7 ETT/TC VAP patients with acute renal failure with VD | AMK sulfate (400 mg/12h) | I-synch VN (connected to the ETT) | BAL in the infection-involved zone | Peak concentration on day 3: 887 (406-12,819) µg/mL | 19 |
| Lu et al. (2011) [20] | In vitro | 46 ETT VAP patients with VD (24 *vs* 22) | AMK + Ceftazidime | C-O VN (10 cm of the Y-piece) | WR of the ventilator circuit | Dose reaching the inlet of the ETT: 63% ND | 21 |
| Niederman et al. (2012) [21] | Randomized comparative | 34 ETT VAP patients with VD (14 *vs* 20) | AMK sulfate (400 mg/12h *vs* 400 mg/24h) | I-synch VN (connected to the ETT) | ETA | Similar peak concentration on day 1: 11,903 ± 11,784 *vs* 6,083 ± 3,528 µg/mL | 26 |
| Athanassa et al. (2012) [22] | Cohort | 20 ETT VAT patients with VD | CMS (80 mg) | C-O VN (15 cm of the Y-piece) | Blind Mini-BAL | Peak concentration on day 1: 6.73 (4.8-10.1) µg/mL | 20 |

**Table S1 (7/8).** Characteristics and results of the clinical studies (Continuation)

| **Study (Year) (Ref)** | **Design** | **Population, artificial airways (ETT/TC)** | **Drug(s) or tracer** | **Administration modality (position)** | **Lung deposition analysis** | **Outcomes of interest** | **D&B score (/28)** |
| --- | --- | --- | --- | --- | --- | --- | --- |
| Montgomery et al. (2014) [23] | Cohort | 7 ETT VAT or VAP patients with VD | AMK (300 mg) and Fosfomycin (120 mg) | C-O VN (15 cm of the Y-piece) | ETA | - Peak concentration of amikacin: 12,390 ± 3,986 µg/mL - Peak concentration of fosfomycin: 6,174 ± 2,548 µg/mL | 21 |
| Boisson et al. (2014) [24] | Comparative cross-over | 12 ETT VAP patients with VD | CMS (160 mg) | C-O VN (NR) *vs* Iv admin | Blind Mini-BAL | Concentration: from 9.53 to 1,137 µg/mL *vs* 1.48 to 28.9 µg/mL* | 19 |
| Lee et al. (2015) [25] | Cohort | 20 ETT patients undergoing abdominal surgery, with VD | Ipratropium bromide (0.5 mg) | C-O VN *vs* C-O JN (connected to the ETT using a horizontal T-piece) | Plasma PK (4 hours) | - Peak concentration: 263.9 ± 63.5 *vs* 313.4 ± 103.0 pg/mL* - AUC normalized to the emitted dose (exiting the T-piece) was similar with both nebulizers | 19 |

**Table S1 (8/8).** Characteristics and results of the clinical studies (Continuation)

| **Study (Year) (Ref)** | **Design** | **Population, artificial airways (ETT/TC)** | **Drug(s)** | **Administration modality (position)** | **Lung deposition analysis** | **Outcomes of interest** | **D&B score (/28)** |
| --- | --- | --- | --- | --- | --- | --- | --- |
| Dugernier et al. (2016) [26] | Randomized Comparative | 17 ETT patients after neurosurgery (9 in VCV *vs* 8 in PSV) | 99mTc-DTPA | C-O VN (connected to the ETT) | γ-Scinti (planar) | - WLD: 15.1 ± 5.0 *vs* 10.5 ± 3.0 % ND* - Similar RLD/LLD ratio: 3.33 (0.7-5.38) *vs* 1.39 (0.91-2.05) - Similar O/I with both modes - Similar O/I for the RL and LL: 0.32 (0.16-0.77) *vs* 0.74 (0.6-1.06) in VCV and 0.75 (0.30-0.94) *vs* 0.67 (0.53-0.86) in PSV | 25 |

Data expressed as mean ± standard deviation, median (25-75% IQR).

%ID, percentage of inhaled dose; % ND, percentage of nominal dose; γ-Scinti, gamma scintigraphy; AMK, amikacin; AUC, area under the curve; BAL, bronchoalveolar lavage; C-O, constant-output; CMS, colistimethate sodium ; DTPA, diethylenetriaminepentaacetic acid ; ETA, endotracheal aspiration; ETT, endotracheal tube; JN, jet nebulizer; I, inner lung region ; I-synch, inspiratory synchronized; Iv, intravenous; LLD, left lung deposition; LTS, lung tissue sampling; MDI, metered-dose inhaler; NR, not reported; O, outer lung region ; PK, pharmacokinetics; PSV, pressure support ventilation; RCT, randomized controlled trial; RLD, right lung deposition; SPECT-CT, single photon emission tomography combined to a CT-scan; TC, tracheostomy cannula; UN, ultrasonic nebulizer; VAP, ventilation-associated pneumonia; VAT, ventilation-associated tracheobronchitis; VCV, volume controlled-ventilation; VD, various diagnoses; VN, vibrating-mesh nebulizer; WLD, whole lung deposition; WR, water rinsing.

* p < 0.001 for the compared data

^†^ p < 0.05 for the compared data

**Table S2 (1/5)**. Characteristics and results of the experimental studies

| **Study (Year) (Ref)** | **Design** | **Population** | **Drug or tracer** | **Administration modality (position)** | **Lung deposition analysis** | **Outcomes related to lung deposition** | **D&B score (/28)** |
| --- | --- | --- | --- | --- | --- | --- | --- |
| Goldstein et al. (2002) [27] | Comparative | 36 ETT Healthy Piglets (18 *vs* 18) | AMK | C-O UN (40 cm of the Y-piece) *vs* Iv admin | - WR of the ventilator circuit and the ETT - LTS of subpleural regions | - ID: 38% ND - Peak concentration in dependent region of the lower lobe: 208 ± 76 *vs* 13 ± 10 µg/g* - Similar concentrations in the RL and LL, in perihilar/subpleural regions, in dep/nondependent regions of the lower lobe) | 19 |
| Elman et al. (2002) [28] | Comparative | 18 ETT VAP Piglets | AMK | C-O UN (40 cm of the Y-piece) *vs* Iv admin | - WR of the ventilator circuit and the ETT - LTS (1h after admin) of subpleural regions with BPN | - ID: 38% ND - Concentration in mild BPN: 197 ± 165 *vs* 6 ± 5 µg/g^†^ - Concentration in severe BPN: 40 ± 62 *vs* 5 ± 3 µg/g* - Higher peak concentration in mild *vs* severe BPN* | 19 |

**Table S2 (2/5)**. Characteristics and results of the experimental studies (Continuation)

| **Study (Year) (Ref)** | **Design** | **Population** | **Drug or tracer** | **Administration modality (position)** | **Lung deposition analysis** | **Outcomes related to lung deposition** | **D&B score (/28)** |
| --- | --- | --- | --- | --- | --- | --- | --- |
| Goldstein et al. (2002) [29] | Comparative | 18 ETT VAP Piglets (10 *vs* 8) | AMK | C-O UN (40 cm of the Y-piece) *vs* Iv admin | - WR of the ventilator circuit and the ETT - LTS of subpleural regions with BPN | - ID: 38% ND - Peak concentration in mild BPN: 188 ± 175 *vs* 5.5 ± 4.5 µg/g^†^ - Peak concentration in severe BPN: 40 ± 65 *vs* 5.1 ± 2.9 µg/g^†^ - Higher peak concentration in mild *vs* severe BPN^†^ | 19 |
| Ferrari et al. (2003) [30] | Comparative | 13 ETT Healthy Piglets (4 *vs* 9) | AMK | C-O UN (40 cm of the Y-piece) after 28h *vs* 75h of MV | LTS of subpleural regions of the lower lobe | - Similar concentration (1h after admin) in dependent and nondependent regions after 28h of MV: 50 to 400 µg/g - Similar concentration in nondependent regions after 75h of MV but lower concentration in dependent regions: 20 to 60 µg/g* | 19 |

**Table S2 (3/5)**. Characteristics and results of the experimental studies (Continuation)

| **Study (Year) (Ref)** | **Design** | **Population** | **Drug or tracer** | **Administration modality (position)** | **Lung deposition analysis** | **Outcomes related to lung deposition** | **D&B score (/28)** |
| --- | --- | --- | --- | --- | --- | --- | --- |
| Tonnelier et al. (2005) [31] | Comparative | 15 ETT Healthy Piglets (5 *vs* 5 *vs* 5) and 15 VAP Piglets (5 *vs* 5 *vs* 5) | Ceftazidime | C-O UN (40 cm of the Y-piece) with He-O_2_ *vs* UN with N_2_-O_2_ *vs* Iv admin | - WR of the ventilator circuit and the ETT - LTS | - Similar inhaled dose with He-O_2_ or N_2_-O_2_: 38% ND - Peak concentration in healthy segments: 576 ± 141 *vs* 383 ± 84 *vs* 17 ± 13 µg/g   * between groups   - Similar peak concentration in infected segments : 111 ± 104 *vs* 129 ± 108 *vs* 10 ± 3 µg/g   ^†^ N_2_-O_2_ *vs* Iv | 19 |
| Ferrari et al. (2008) [32] | Comparative | 10 ETT Healthy Piglets (5 *vs* 5) | Ceftazidime | C-O VN (15 cm of the Y-piece) *vs* C-O UN (40 cm of the Y-piece) | - WR of the ventilator circuit and the ETT - LTS of subpleural regions | - Similar inhaled dose: 62 ± 5% vs 66 ± 4% ND with 50 and 10% ND in proximal and distal airways - Similar peak concentration in healthy segments: 452 ± 172 *vs* 553 ± 123 µg/g | 18 |

**Table S2 (4/5)**. Characteristics and results of the experimental studies (Continuation)

| **Study (Year) (Ref)** | **Design** | **Population** | **Drug or tracer** | **Administration modality (position)** | **Lung deposition analysis** | **Outcomes related to lung deposition** | **D&B score (/28)** |
| --- | --- | --- | --- | --- | --- | --- | --- |
| Selting et al. (2008) [33] | Comparative | 6 ETT Dogs (3 *vs* 3) | Cisplatin | Intracorporeal nebulizing catheter in the RCLL *vs* Iv | LTS | 44-times more cisplatin (values NR) in RCLL with the nebulizing catheter* | 18 |
| Ferrari et al. (2009) [34] | Comparative | 12 ETT VAP Piglets (6 vs 6) | Ceftazidime | C-O VN (15 cm of the Y-piece) *vs* Iv | - WR of the ventilator circuit and the ETT - LTS of subpleural regions with BPN | - ID: 40.7 ± 7.1% ND - Through concentration in infected specimens: 24.8 (12.6-59.6) *vs* 6.1 (4.6-10.8) µg/g* - Higher through concentration in mild *vs* severe BPN^†^ | 18 |
| Lu et al. (2010) [35] | Comparative | 12 ETT VAP Piglets (6 *vs* 6) | Colistin | Continuous VN (15 cm of the Y-piece) *vs* Iv | - WR of the ventilator circuit and the ETT - LTS of subpleural regions with BPN | - ID: 60% ND (50% in the tracheobronchial area and 10% in subpleural specimens) - Peak concentration: 2.8 (0.8-13.7) µg/g *vs* Not detectable - Higher concentration in mild *vs* severe BPN^†^ | 18 |

**Table S2 (5/5)**. Characteristics and results of the experimental studies (Continuation)

| **Study (Year) (Ref)** | **Design** | **Population** | **Drug or tracer** | **Administration modality (position)** | **Lung deposition analysis** | **Outcomes related to lung deposition** | **D&B score (/28)** |
| --- | --- | --- | --- | --- | --- | --- | --- |
| Guillon et al. (2015) [36] | Cohort | 6 ETT Healthy Pigs | Teicoplanin labeled with 99mTc-DTPA | Continuous VN (insp limb, location NR) | γ-Scinti (planar) | - ID: 23.3 ± 7.2% ND - Non normalized O/I deposition ratio = 2^‡^ | 18 |

Data expressed as mean ± standard deviation, median (25-75 % IQR). Lung tissue sampling was performed in lung regions with bronchopneumonia classified as mild (bronchiolitis, small foci of pneumonia, interstitial pneumonia) or severe (confluent pneumonia, necrotizing pneumonia) [28].

% ND, percentage of nominal dose; γ-Scinti, gamma scintigraphy; AMK, amikacin; BPN, bronchopneumonia; C-O, constant-output; DTPA, diethylenetriaminepentaacetic acid; ETT, endotracheal tube; I, inner lung region ; I-synch, inspiratory synchronized; Iv, intravenous; LLD, left lung deposition; LTS, lung tissue sampling; MV, mechanical ventilation; NR, not reported; O, outer lung region ; PK, pharmacokinetics; PSV, pressure support ventilation; RCLL, right caudal lung lobe ; RCT, randomized controlled trial; RLD, right lung deposition; SPECT-CT, single photon emission tomography combined to a CT-scan; UN, ultrasonic nebulizer; VAP, ventilation-associated pneumonia; VAT, ventilation-associated tracheobronchitis; VCV, volume controlled-ventilation; VN, vibrating-mesh nebulizer; WR, water rinsing.

* p < 0.001 for the compared data

^†^ p < 0.05 for the compared data

^‡^ The O/I ratio of 2 reported in ventilated piglets was not normalized to the lung volume as recommended in international guidelines [37] but the O and I region differ in relative lung area and thickness. Hence, the non-normalized O/I ratio tend to overestimate the penetration index [38].

**Table S3 (1/3).** Downs and Black score of the included studies

| **Author (years) (Ref)** | **Reporting (/10)** | **External validity (/3)** | **Internal validity-bias (/7)** | **Internal validity-(confounding) selection bias (/6)** | **Power**  **(/1)** |
| --- | --- | --- | --- | --- | --- |
| MacIntyre et al. (1985) [1] | 9 | 3 | 5 | 3 | 0 |
| Girard et al. (1989) [2] | 8 | 3 | 4 | 3 | 0 |
| Fuller et al. (1990) [3] | 9 | 3 | 5 | 4 | 0 |
| Le Conte et al. (1992) [4] | 7 | 3 | 5 | 3 | 0 |
| Thomas et al. (1993) [5] | 10 | 3 | 5 | 3 | 0 |
| O'Riordan et al. (1994) [6] | 10 | 3 | 4 | 3 | 0 |
| Fuller et al. (1994) [7] | 9 | 3 | 5 | 4 | 1 |
| Harvey et al. (1995) [8] | 9 | 3 | 5 | 3 | 0 |
| Duarte et al. (1996) [9] | 10 | 3 | 5 | 3 | 0 |
| Harvey et al. (1997) [10] | 9 | 3 | 5 | 3 | 0 |
| Palmer et al. (1998) [11] | 10 | 3 | 5 | 4 | 0 |
| Marik et al. (1999) [12] | 10 | 3 | 5 | 4 | 0 |
| Wood et al. (2000) [13] | 8 | 3 | 7 | 3 | 1 |
| Goldstein et al. (2002) [27] | 10 | 2 | 5 | 3 | 0 |
| Elman et al. (2002) [28] | 10 | 2 | 5 | 3 | 0 |

**Table S3 (2/3).** Downs and Black score of the included studies (Continuation)

| **Author (years) (Ref)** | **Reporting (/10)** | **External validity (/3)** | **Internal validity-bias (/7)** | **Internal validity-(confounding) selection bias (/6)** | **Power**  **(/1)** |
| --- | --- | --- | --- | --- | --- |
| Goldstein et al. (2002) [29] | 10 | 2 | 5 | 3 | 0 |
| Miller et al. (2003) [14] | 8 | 3 | 5 | 3 | 0 |
| Ferrari et al. (2003) [30] | 10 | 2 | 5 | 3 | 0 |
| Badia et al. (2004) [15] | 10 | 3 | 5 | 5 | 1 |
| Klockare et al. (2005) [16] | 8 | 3 | 5 | 3 | 1 |
| Tonnellier et al. (2005) [31] | 10 | 2 | 5 | 3 | 0 |
| Ferrari et al. (2008) [32] | 9 | 2 | 5 | 3 | 0 |
| Selting et al. (2008) [33] | 9 | 2 | 5 | 3 | 0 |
| Ferrari et al. (2009) [34] | 9 | 2 | 5 | 3 | 0 |
| Moraine et al. (2009) [17] | 9 | 3 | 5 | 4 | 0 |
| Luyt et al. (2009) [18] | 9 | 3 | 5 | 3 | 0 |
| Lu et al. (2010) [35] | 9 | 2 | 5 | 3 | 0 |
| Lu et al. (2011) [20] | 11 | 3 | 5 | 3 | 0 |
| Luyt et al. (2011) [19] | 9 | 3 | 5 | 3 | 0 |
| Niederman et al. (2012) [21] | 10 | 3 | 7 | 6 | 1 |
| Athanassa et al. (2012) [22] | 10 | 3 | 5 | 3 | 0 |

**Table S3 (3/3).** Downs and Black score of the included studies (Continuation)

| **Author (years) (Ref)** | **Reporting (/10)** | **External validity (/3)** | **Internal validity-bias (/7)** | **Internal validity-(confounding) selection bias (/6)** | **Power**  **(/1)** |
| --- | --- | --- | --- | --- | --- |
| Montgomery et al. (2014) [23] | 9 | 3 | 7 | 3 | 0 |
| Boisson et al. (2014) [24] | 8 | 3 | 5 | 4 | 0 |
| Guillon et al. (2015) [36] | 9 | 2 | 5 | 3 | 0 |
| Lee et al. (2016) [25] | 8 | 3 | 5 | 4 | 0 |
| Dugernier et al. (2016) [26] | 10 | 3 | 7 | 5 | 1 |

**Table S4 (1/5).** Mechanical ventilation characteristics during inhalation

| **Study (Year)** | **Mode** | **V_T_**  **(mL)** | **RR (c/min)** | **T_insp_/T_tot_**  **(%)** | **Flow _insp_ (L/min)** | **Insp pause (%)** | **PEEP (cmH2O)** | **FiO_2_**  **(%)** | **Bias Flow (L/min)** | **H-Humidif** | **Inspired gas** |
| --- | --- | --- | --- | --- | --- | --- | --- | --- | --- | --- | --- |
| MacIntyre et al. (1985) [1] | PCV or VCV | 800-1200 | 6-12 | NR | NR | NR | 0 to 15 | NR | NR | NR | N_2_-O_2_ |
| Girard et al. (1989) [2] | NR | NR | NR | NR | NR | NR | 0 to 18 | NR | NR | Yes | N_2_-O_2_ |
| Fuller et al. (1990) [3] | VAC | 400-800 | NR | NR | NR | NR | NR | NR | NR | No | N_2_-O_2_ |
| Le Conte et al. (1993) [4] | VCV | 750 | 16 | NR, controlled | NR | NR | NR | < 40 | NR | NR | N_2_-O_2_ |
| Thomas et al. (1993) [5] | VCV | NR | 13  (12-15) | 30 (25-33) | NR | 17  (10-20) | NR | 59  (30-80) | NR | Yes | N_2_-O_2_ |
| O’Riordan et al. (1994) [6] | VCV | 500-1000 | 10-16 | 25 ± 9 | 40 to 50 | NR | NR | NR | NR | No | N_2_-O_2_ |
| Fuller et al. (1994) [7] | VAC | NR | NR | NR | NR | NR | NR | NR | NR | Yes | N_2_-O_2_ |

**Table S4 (2/5).** Mechanical ventilation characteristics during inhalation (Continuation)

| **Study (Year)** | **Mode** | **V_T_**  **(mL)** | **RR (c/min)** | **T_insp_/T_tot_**  **(%)** | **Flow _insp_ (L/min)** | **Insp pause (%)** | **PEEP (cmH2O)** | **FiO_2_**  **(%)** | **Bias Flow (L/min)** | **H-Humidif** | **Inspired gas** |
| --- | --- | --- | --- | --- | --- | --- | --- | --- | --- | --- | --- |
| Harvey et al. (1995) [8] | VCV | NR | 15  (14-16) | 26  (25-33) | NR | 11  (10-20) | NR | NR | NR | Yes | N_2_-O_2_ |
| Duarte et al. (1996) [9] | VCV | 500-800 | 12-20 | 14-20 | Constant, NR | No | NR | 30-50 | NR | Yes | N_2_-O_2_ |
| Harvey et al. (1997) [10] | VCV | NR | 15  (14-16) | 24  (15-25) | NR | 10 | NR | NR | NR | Yes | N_2_-O_2_ |
| Palmer et al. (1998) [11] | VCV | 600-1000 | 10-15 | NR | Constant, 40 | NR | NR | NR | NR | No | N_2_-O_2_ |
| Marik et al. (1999) [12] | VCV | 590-750 | 13 | >30 | NR | NR | 4-5 | NR | Off | NR | N_2_-O_2_ |
| Goldstein et al. (2002) [27] | VCV | 300  (15 mL/kg) | 15 | 50 | Constant, 11 | 20 | 0 | 21 | NR | No | N_2_-O_2_ |
| Wood et al. (2002) [13] | NR | NR | NR | NR | NR | NR | NR | NR | NR | Off | N_2_-O_2_ |
| Elman et al. (2002) [28] | VCV | 300  (15 mL/kg) | 15 | 50 | Constant, 11 | 20 | 0 | 21 | NR | No | N_2_-O_2_ |

**Table S4 (3/5).** Mechanical ventilation characteristics during inhalation (Continuation)

| **Study (Year)** | **Mode** | **V_T_**  **(mL)** | **RR (c/min)** | **T_insp_/T_tot_**  **(%)** | **Flow _insp_ (L/min)** | **Insp pause (%)** | **PEEP (cmH2O)** | **FiO_2_**  **(%)** | **Bias Flow (L/min)** | **H-Humidif** | **Inspired gas** |
| --- | --- | --- | --- | --- | --- | --- | --- | --- | --- | --- | --- |
| Goldstein et al. (2002) [29] | VCV | 300  (15 mL/Kg) | 15 | 50 | Constant, 11 | 20 | 0 | 21 | NR | No | N_2_-O_2_ |
| Ferrari et al. (2003) [30] | VCV | 300  (15 mL/Kg) | 15 | 50 | Constant, 11 | 20 | 0 | 21 | NR | No | N_2_-O_2_ |
| Miller et al. (2003) [14] | VAC or SIMV | 450-700 | 12-18 | NR | NR | NR | NR | 30-50 | NR | Yes *vs* No | N_2_-O_2_ |
| Badia et al. (2004) [15] | VCV | NR (8-10 mL/Kg) | NR | NR | NR | NR | 0 | NR | NR | No | N_2_-O_2_ |
| Klockare et al. (2005) [16] | VCV and PSV | 530-830 | 12-24 | NR | NR | NR | NR | 30-45 | NR | NR | N_2_-O_2_ |
| Tonnelier et al. (2005) [31] | VCV | 300  (15 mL/Kg) | 15 | 50 | Constant, 11 | 20 | 5 | 21 | NR | No | N_2_-O_2_ *vs* He-O_2_ |
| Selting et al. (2008) [33] | VCV | NR | NR | NR | NR | NR | NR | NR | NR | NR | N_2_-O_2_ |

**Table S4 (4/5).** Mechanical ventilation characteristics during inhalation (Continuation)

| **Study (Year) (Ref)** | **Mode** | **V_T_**  **(mL)** | **RR (c/min)** | **T_insp_/T_tot_**  **(%)** | **Flow _insp_ (L/min)** | **Insp pause (%)** | **PEEP (cmH2O)** | **FiO_2_**  **(%)** | **Bias Flow (L/min)** | **H-Humidif** | **Inspired gas** |
| --- | --- | --- | --- | --- | --- | --- | --- | --- | --- | --- | --- |
| Ferrari et al. (2008) [32] | VCV | 300  (15 mL/Kg) | 15 | 50 | Constant, 11 | 20 | 5 | 21 | NR | No | He-O_2_ |
| Ferrari et al. (2009) [34] | VCV | 300  (15 mL/Kg) | 15 | 50 | Constant, 11 | 20 | 5 | 21 | NR | No | N_2_-O_2_ |
| Moraine et al. (2009) [17] | VCV | 600 | 12-13 | NR | NR | NR | NR | NR | NR | Off | N_2_-O_2_ |
| Luyt et al. (2009) [18] | PCV or VAC | NR | NR | NR | NR | NR | NR | NR | NR | Yes | N_2_-O_2_ |
| Lu et al. (2010) [35] | VCV | 300  (15 mL/Kg) | 15 | 50 | Constant, 11 | 20 | 5 | 21 | NR | No | N_2_-O_2_ |
| Luyt et al. (2011) [19] | PCV or VAC | NR | NR | NR | NR | NR | NR | NR | NR | Yes | N_2_-O_2_ |
| Lu et al. (2011) [20] | VCV | NR  (8 mL/Kg) | 12 | 50 | Constant, NR | 20 | NR | NR | NR | No | N_2_-O_2_ |

**Table S4 (5/5).** Mechanical ventilation characteristics during inhalation (Continuation)

| **Study (Year) (Ref)** | **Mode** | **V_T_**  **(mL)** | **RR (c/min)** | **T_insp_/T_tot_**  **(%)** | **Flow _insp_ (L/min)** | **Insp pause (%)** | **PEEP (cmH2O)** | **FiO_2_**  **(%)** | **Bias Flow (L/min)** | **H-Humidif** | **Inspired gas** |
| --- | --- | --- | --- | --- | --- | --- | --- | --- | --- | --- | --- |
| Niederman et al. (2012) [21] | VAC or PSV | NR | NR | NR | NR | NR | NR | NR | NR | Yes | N_2_-O_2_ |
| Athanassa et al. (2012) [22] | VAC | 500-600 | 12-14 | 50 | Constant, NR | NR | 5 | NR | NR | No | N_2_-O_2_ |
| Montgomery et al. (2014) [23] | NR,  SB mode | 551 ± 54 | NR | NR | NR | NR | 5-12.5 | 30-50 | ≤ 2 | Yes | N_2_-O_2_ |
| Boisson et al. (2014) [24] | NR | NR | NR | NR | NR | NR | NR | NR | NR | NR | N_2_-O_2_ |
| Guillon et al. (2015) [36] | VCV | NR (8-10 mL/Kg) | 15 | 50 | Constant | NR | 5 | 50 | NR | NR | N_2_-O_2_ |
| Lee et al. (2016) [25] | VCV | NR  (8 mL/Kg) | 10 | 50 | NR | NR | NR | NR | NR | NR | N_2_-O_2_ |
| Dugernier et al. (2016) [26] | VCV *vs* PSV | 530 ± 95 *vs* 585 ± 117  (8 mL/Kg) | 18 ± 2 *vs*  14 ± 1 | 32 *vs* 25 | 32 ± 4 *vs* 44 ± 8 | No *vs* NA | 5 | NR | 10 | No | N_2_-O_2_ |

Data expressed as mean ± standard deviation, median (25-75) interquartile range or (minimum-maximum). FiO_2_, inspired fraction of oxygen; NA, not applicable; NR, not reported; PSV, pressure support ventilation; RR, respiratory rate; VAC, volume assist control ventilation; VCV, volume-controlled ventilation; V_T_, tidal volume.

References Table S1, S2, S3 and S4

1. MacIntyre NR, Silver RM, Miller CW, Schuler F, Coleman RE, (1985) Aerosol delivery in intubated, mechanically ventilated patients. Crit Care Med 13: 81-84

2. Girard PM, Clair B, Certain A, Bidault R, Matheron S, Regnier B, Farinotti R, (1989) Comparison of plasma concentrations of aerosolized pentamidine in nonventilated and ventilated patients with pneumocystosis. Am Rev Respir Dis 140: 1607-1610

3. Fuller HD, Dolovich MB, Posmituck G, Pack WW, Newhouse MT, (1990) Pressurized aerosol versus jet aerosol delivery to mechanically ventilated patients. Comparison of dose to the lungs. Am Rev Respir Dis 141: 440-444

4. Le Conte P, Potel G, Peltier P, Horeau D, Caillon J, Juvin ME, Kergueris MF, Bugnon D, Baron D, (1993) Lung distribution and pharmacokinetics of aerosolized tobramycin. Am Rev Respir Dis 147: 1279-1282

5. Thomas SH, O'Doherty MJ, Fidler HM, Page CJ, Treacher DF, Nunan TO, (1993) Pulmonary deposition of a nebulised aerosol during mechanical ventilation. Thorax 48: 154-159

6. O'Riordan TG, Palmer LB, Smaldone GC, (1994) Aerosol deposition in mechanically ventilated patients. Optimizing nebulizer delivery. Am J Respir Crit Care Med 149: 214-219

7. Fuller HD, Dolovich MB, Turpie FH, Newhouse MT, (1994) Efficiency of bronchodilator aerosol delivery to the lungs from the metered dose inhaler in mechanically ventilated patients. A study comparing four different actuator devices. Chest 105: 214-218

8. Harvey CJ, O'Doherty MJ, Page CJ, Thomas SH, Nunan TO, Treacher DF, (1995) Effect of a spacer on pulmonary aerosol deposition from a jet nebuliser during mechanical ventilation. Thorax 50: 50-53

9. Duarte AG, Dhand R, Reid R, Fink JB, Fahey PJ, Tobin MJ, Jenne JW, (1996) Serum albuterol levels in mechanically ventilated patients and healthy subjects after metered-dose inhaler administration. Am J Respir Crit Care Med 154: 1658-1663

10. Harvey CJ, O'Doherty MJ, Page CJ, Thomas SH, Nunan TO, Treacher DF, (1997) Comparison of jet and ultrasonic nebulizer pulmonary aerosol deposition during mechanical ventilation. The European respiratory journal 10: 905-909

11. Palmer LB, Smaldone GC, Simon SR, O'Riordan TG, Cuccia A, (1998) Aerosolized antibiotics in mechanically ventilated patients: delivery and response. Crit Care Med 26: 31-39

12. Marik P, Hogan J, Krikorian J, (1999) A comparison of bronchodilator therapy delivered by nebulization and metered-dose inhaler in mechanically ventilated patients. Chest 115: 1653-1657

13. Wood GC, Boucher BA, Croce MA, Hanes SD, Herring VL, Fabian TC, (2002) Aerosolized ceftazidime for prevention of ventilator-associated pneumonia and drug effects on the proinflammatory response in critically ill trauma patients. Pharmacotherapy 22: 972-982

14. Miller DD, Amin MM, Palmer LB, Shah AR, Smaldone GC, (2003) Aerosol delivery and modern mechanical ventilation: in vitro/in vivo evaluation. Am J Respir Crit Care Med 168: 1205-1209

15. Badia JR, Soy D, Adrover M, Ferrer M, Sarasa M, Alarcon A, Codina C, Torres A, (2004) Disposition of instilled versus nebulized tobramycin and imipenem in ventilated intensive care unit (ICU) patients. J Antimicrob Chemother 54: 508-514

16. Klockare M, Dufva A, Danielsson AM, Hatherly R, Larsson S, Jacobsson H, Mure M, (2006) Comparison between direct humidification and nebulization of the respiratory tract at mechanical ventilation: distribution of saline solution studied by gamma camera. J Clin Nurs 15: 301-307

17. Moraine JJ, Truflandier K, Vandenbergen N, Berre J, Melot C, Vincent JL, (2009) Placement of the nebulizer before the humidifier during mechanical ventilation: Effect on aerosol delivery. Heart Lung 38: 435-439

18. Luyt CE, Clavel M, Guntupalli K, Johannigman J, Kennedy JI, Wood C, Corkery K, Gribben D, Chastre J, (2009) Pharmacokinetics and lung delivery of PDDS-aerosolized amikacin (NKTR-061) in intubated and mechanically ventilated patients with nosocomial pneumonia. Crit Care 13: R200

19. Luyt CE, Eldon MA, Stass H, Gribben D, Corkery K, Chastre J, (2011) Pharmacokinetics and tolerability of amikacin administered as BAY41-6551 aerosol in mechanically ventilated patients with gram-negative pneumonia and acute renal failure. J Aerosol Med Pulm Drug Deliv 24: 183-190

20. Lu Q, Yang J, Liu Z, Gutierrez C, Aymard G, Rouby JJ, Nebulized Antibiotics Study G, (2011) Nebulized ceftazidime and amikacin in ventilator-associated pneumonia caused by Pseudomonas aeruginosa. Am J Respir Crit Care Med 184: 106-115

21. Niederman MS, Chastre J, Corkery K, Fink JB, Luyt CE, Garcia MS, (2012) BAY41-6551 achieves bactericidal tracheal aspirate amikacin concentrations in mechanically ventilated patients with Gram-negative pneumonia. Intensive Care Med 38: 263-271

22. Athanassa ZE, Markantonis SL, Fousteri MZ, Myrianthefs PM, Boutzouka EG, Tsakris A, Baltopoulos GJ, (2012) Pharmacokinetics of inhaled colistimethate sodium (CMS) in mechanically ventilated critically ill patients. Intensive Care Med 38: 1779-1786

23. Montgomery AB, Vallance S, Abuan T, Tservistas M, Davies A, (2014) A randomized double-blind placebo-controlled dose-escalation phase 1 study of aerosolized amikacin and fosfomycin delivered via the PARI investigational eFlow(R) inline nebulizer system in mechanically ventilated patients. J Aerosol Med Pulm Drug Deliv 27: 441-448

24. Boisson M, Jacobs M, Gregoire N, Gobin P, Marchand S, Couet W, Mimoz O, (2014) Comparison of intrapulmonary and systemic pharmacokinetics of colistin methanesulfonate (CMS) and colistin after aerosol delivery and intravenous administration of CMS in critically ill patients. Antimicrob Agents Chemother 58: 7331-7339

25. Lee YH, Kwon GY, Park DY, Bang JY, Jang DM, Lee SH, Lee EK, Choi BM, Noh GJ, (2016) Efficiency of a New Mesh-Type Nebulizer (NE-SM1 NEPLUS) for Intrapulmonary Delivery of Ipratropium Bromide in Surgical Patients. Basic Clin Pharmacol Toxicol 118: 313-319

26. Dugernier J, Reychler G, Wittebole X, Roeseler J, Depoortere V, Sottiaux T, Michotte JB, Vanbever R, Dugernier T, Goffette P, Docquier MA, Raftopoulos C, Hantson P, Jamar F, Laterre PF, (2016) Aerosol delivery with two ventilation modes during mechanical ventilation: a randomized study. Ann Intensive Care 6: 73

27. Goldstein I, Wallet F, Robert J, Becquemin MH, Marquette CH, Rouby JJ, (2002) Lung tissue concentrations of nebulized amikacin during mechanical ventilation in piglets with healthy lungs. Am J Respir Crit Care Med 165: 171-175

28. Elman M, Goldstein I, Marquette CH, Wallet F, Lenaour G, Rouby JJ, Experimental ICUSG, (2002) Influence of lung aeration on pulmonary concentrations of nebulized and intravenous amikacin in ventilated piglets with severe bronchopneumonia. Anesthesiology 97: 199-206

29. Goldstein I, Wallet F, Nicolas-Robin A, Ferrari F, Marquette CH, Rouby JJ, (2002) Lung deposition and efficiency of nebulized amikacin during Escherichia coli pneumonia in ventilated piglets. Am J Respir Crit Care Med 166: 1375-1381

30. Ferrari F, Goldstein I, Nieszkowszka A, Elman M, Marquette CH, Rouby JJ, Experimental ICUSG, (2003) Lack of lung tissue and systemic accumulation after consecutive daily aerosols of amikacin in ventilated piglets with healthy lungs. Anesthesiology 98: 1016-1019

31. Tonnellier M, Ferrari F, Goldstein I, Sartorius A, Marquette CH, Rouby JJ, (2005) Intravenous versus nebulized ceftazidime in ventilated piglets with and without experimental bronchopneumonia: comparative effects of helium and nitrogen. Anesthesiology 102: 995-1000

32. Ferrari F, Liu ZH, Lu Q, Becquemin MH, Louchahi K, Aymard G, Marquette CH, Rouby JJ, (2008) Comparison of lung tissue concentrations of nebulized ceftazidime in ventilated piglets: ultrasonic versus vibrating plate nebulizers. Intensive Care Med 34: 1718-1723

33. Selting K, Waldrep JC, Reinero C, Branson K, Gustafson D, Kim DY, Henry C, Owen N, Madsen R, Dhand R, (2008) Feasibility and safety of targeted cisplatin delivery to a select lung lobe in dogs via the AeroProbe intracorporeal nebulization catheter. J Aerosol Med Pulm Drug Deliv 21: 255-268

34. Ferrari F, Lu Q, Girardi C, Petitjean O, Marquette CH, Wallet F, Rouby JJ, Experimental ICUSG, (2009) Nebulized ceftazidime in experimental pneumonia caused by partially resistant Pseudomonas aeruginosa. Intensive Care Med 35: 1792-1800

35. Lu Q, Girardi C, Zhang M, Bouhemad B, Louchahi K, Petitjean O, Wallet F, Becquemin MH, Le Naour G, Marquette CH, Rouby JJ, (2010) Nebulized and intravenous colistin in experimental pneumonia caused by Pseudomonas aeruginosa. Intensive Care Med 36: 1147-1155

36. Guillon A, Mercier E, Lanotte P, Haguenoer E, Darrouzain F, Barc C, Sarradin P, Si-Tahar M, Heuze-Vourc'h N, Diot P, Vecellio L, (2015) Aerosol Route to Administer Teicoplanin in Mechanical Ventilation: In Vitro Study, Lung Deposition and Pharmacokinetic Analyses in Pigs. J Aerosol Med Pulm Drug Deliv 28: 290-298

37. Newman S, Bennett WD, Biddiscombe M, Devadason SG, Dolovich MB, Fleming J, Haeussermann S, Kietzig C, Kuehl PJ, Laube BL, Sommerer K, Taylor G, Usmani OS, Zeman KL, (2012) Standardization of techniques for using planar (2D) imaging for aerosol deposition assessment of orally inhaled products. J Aerosol Med Pulm Drug Deliv 25 Suppl 1: S10-28

38. Biddiscombe MF, Meah SN, Underwood SR, Usmani OS, (2011) Comparing lung regions of interest in gamma scintigraphy for assessing inhaled therapeutic aerosol deposition. J Aerosol Med Pulm Drug Deliv 24: 165-173
